# Temporal trends in spatial inequalities of maternal and newborn health services among four east African countries, 1999–2015

**DOI:** 10.1186/s12889-018-6241-8

**Published:** 2018-12-04

**Authors:** Corrine W. Ruktanonchai, Kristine Nilsen, Victor A. Alegana, Claudio Bosco, Rogers Ayiko, Andrew C. Seven Kajeguka, Zöe Matthews, Andrew J. Tatem

**Affiliations:** 1 WorldPop, Geography and Environmental Science, University of Southampton, Southampton, UK; 2 Flowminder Foundation, Roslagsgatan 17, SE-11355 Stockholm, Sweden; 3 Open Health Initiative, East African Community Secretariat, Arusha, Tanzania; 4 Division of Social Statistics and Demography & Centre for Global Health, Population, Poverty and Policy, University of Southampton, Southampton, UK; 5 Population Health Theme, Kenya Medical Research Institute-Wellcome Trust Research Programme, Nairobi, Kenya; 6 Geography and Environmental Science, University of Southampton, Southampton, SO17 1BJ UK; 7 EAC Integrated Health Programme (EIHP), Health Department, East African Community (EAC) Secretariat, Arusha, United Republic of Tanzania

**Keywords:** Maternal and newborn health, Spatial interpolation, Temporal analysis, East Africa community, Skilled birth attendance, Antenatal care, Postnatal care

## Abstract

**Background:**

Sub-Saharan Africa continues to account for the highest regional maternal mortality ratio (MMR) in the world, at just under 550 maternal deaths per 100,000 live births in 2015, compared to a global rate of 216 deaths. Spatial inequalities in access to life-saving maternal and newborn health (MNH) services persist within sub-Saharan Africa, however, with varied improvement over the past two decades. While previous research within the East African Community (EAC) region has examined utilisation of MNH care as an emergent property of geographic accessibility, no research has examined how these spatial inequalities have evolved over time at similar spatial scales.

**Methods:**

Here, we analysed temporal trends of spatial inequalities in utilisation of antenatal care (ANC), skilled birth attendance (SBA), and postnatal care (PNC) among four East African countries. Specifically, we used Bayesian spatial statistics to generate district-level estimates of these services for several time points using Demographic and Health Surveys data in Kenya, Tanzania, Rwanda, and Uganda. We examined temporal trends of both absolute and relative indices over time, including the absolute difference between estimates, as well as change in performance ratios of the best-to-worst performing districts per country.

**Results:**

Across all countries, we found the greatest spatial equality in ANC, while SBA and PNC tended to have greater spatial variability. In particular, Rwanda represented the only country to consistently increase coverage and reduce spatial inequalities across all services. Conversely, Tanzania had noticeable reductions in ANC coverage throughout most of the country, with some areas experiencing as much as a 55% reduction. Encouragingly, however, we found that performance gaps between districts have generally decreased or remained stably low across all countries, suggesting countries are making improvements to reduce spatial inequalities in these services.

**Conclusions:**

We found that while the region is generally making progress in reducing spatial gaps across districts, improvement in PNC coverage has stagnated, and should be monitored closely over the coming decades. This study is the first to report temporal trends in district-level estimates in MNH services across the EAC region, and these findings establish an important baseline of evidence for the Sustainable Development Goal era.

**Electronic supplementary material:**

The online version of this article (10.1186/s12889-018-6241-8) contains supplementary material, which is available to authorized users.

## Background

Substantial progress has been made in reducing global maternal mortality over the past three decades, and while total coverage of maternal health services may have increased over time, inequalities among those utilising these services have persisted [1, 2]. Sub-Saharan Africa continues to account for the highest regional maternal mortality ratio (MMR) in the world, at just under 550 maternal deaths per 100,000 live births in 2015, compared to a global rate of 216 deaths. Further, only four countries achieved the 75% reduction (Cabo Verde, Equatorial Guinea, Eritrea, and Rwanda) set out by Millennium Development Goal (MDG) 5a [3]. These ratios mask underlying heterogeneity, however, with MMRs ranging from as low as 167 per 100,000 live births in Southern Africa, to as high as 675 deaths per 100,000 live births in Western Africa in 2015 [3]. The greatest reduction in MMR between 1990 and 2015 occurred within the Eastern Africa sub-region (as defined by the United Nations’ (UN) MDG groupings), with a 57% overall change and 3.4% average annual change [4]. However, even within this sub-region, countries falling in the East African Community (EAC) region (comprised in 2015 of Burundi, Kenya, Rwanda, Tanzania, and Uganda, with the addition of South Sudan in 2016) experienced varied improvement in preventing maternal deaths over the past two decades, with Rwanda representing the only country within the region to reach the MDG 5a target.

Reducing health inequalities in low- and middle-income countries has become an increasingly important and quantifiable objective in the post-2015 Sustainable Development Goals (SDG) agenda [5, 6]. While there has been a renewed call for disaggregation of national level indicators, much research in maternal and newborn health (MNH) over the previous decades has focused on disaggregating health data by socioeconomic status such as wealth quintile and education status [1, 4, 7]. The state of a mother’s health depends not only on her education and income, however, but also on where she lives and the progress her country is making in addressing maternal and newborn health issues [4]. Over the coming decades, therefore, spatial and temporal disaggregation in addition to this continued socioeconomic disaggregation will be key to ensuring healthy lives and well-being for all across all ages. By disaggregating data across spatial, temporal and socioeconomic dimensions, health inequalities amongst subgroups may be highlighted, as well as how these inequalities have changed over time [4].

Preventing the deaths of mothers and newborns ultimately requires delivery of timely and high quality service packages and interventions across the continuum of care, such as antenatal care, skilled birth attendance, and postnatal care [8]. Previous studies have examined temporal trends of child mortality [9] and health indicators such as education [10] and child growth [11] at high spatial resolutions, but fewer studies have examined temporal trends in utilisation of key MNH services at similar spatial resolutions. Victora and colleagues [12] reported progress in maternal, newborn, and child survival through the Millennium Development Goal era, while more recently, child and maternal mortality estimates have been systematically reported at the global, regional and national scales [13, 14]. Assaf and Pullum [7] further reported temporal trends in key MNH services disaggregated by socioeconomic indicators such as wealth and education; these studies, however, represent analysis performed at a provincial or national-level spatial scale, and potentially mask important variation at policy-relevant spatial scales, such as the district level.

Within the EAC region, previous research [15] has examined utilisation of MNH services as an emergent property of accessibility, highlighting high-resolution inequalities in receiving care before, during, and after delivery. While these inequalities have been spatially disaggregated, no research has examined how these spatial inequalities have evolved over time at similar spatial scales. Further, estimates at a policy-meaningful level such as the district level remain limited, as data collected in-country tend to be limited by insufficient reporting, sampling bias, or other methodological challenges. Estimates of MNH service utilisation have historically relied on household surveys such as the Demographic and Health Surveys (DHS), but these are not typically representative below the national or regional level due to sample design (with some recent exceptions such as the 2015 Kenya DHS). This necessitates the use of spatial statistics, such as small area estimation techniques and geo-spatial modelling frameworks, to generate predictive estimates at smaller spatial levels, but these approaches also come with methodological considerations. Spatial interpolation methods using a Bayesian framework are one such application of predictive modelling, and represent an ideal opportunity to quantify uncertainty in estimates through posterior distributions [16]. Briefly, a Bayesian framework generates a distribution of possible estimates, or posterior distribution, in which the “true” value may lie, and allows for reporting of standard distribution statistics, such as mean, median, standard deviation, and credibility interval. This is particularly valuable when reporting DHS data at a spatial scale different from that which the data were collected at, where the range of uncertainty may vary in more rural, less sampled areas [16, 17].

Here, this study examines how prevalence of antenatal care (ANC), skilled birth attendance (SBA), and postnatal care (PNC) use has changed sub-nationally over time within the EAC region, using both absolute and relative measures of inequality. Barros and Victora [5] argue that reporting temporal trends of both absolute and relative measures of inequalities is essential, as these measures complement each other to provide a more comprehensive reflection of change in inequality over time. This paper therefore aims to generate estimates of ANC, SBA, and PNC ranging between 1999 and 2015 at a higher spatial resolution than has been previously reported. We examine temporal trends of absolute indices by visualising the difference in these estimates between the first and last time points available. We further aim to examine temporal trends of relative indices by quantifying how the gap between the best-to-worst performing administrative units has changed over time, as well as explore how the effect of space alone has changed in predicting utilisation of these services.

## Methods

### Data

To explore sub-national change in ANC, SBA, and PNC over time, we compiled data from DHS for Kenya, Tanzania, Rwanda, and Uganda for several time points available (see Table 1) using SAS version 9.4 software [18]. To calculate estimates using DHS data at a spatial area smaller than those which are reported through the DHS program, information on spatial location of household surveys are necessary through global positioning system (GPS) coordinates [16]. The DHS program provides this information for recent surveys at the cluster (an aggregate of households) level, which is then displaced to maintain participant confidentiality. To facilitate spatial interpolation, we therefore included only standard DHS surveys in these analyses with corresponding geo-located cluster data available. Of note, at the time these analyses were performed, Burundi contained a full DHS survey with associated GPS data for only 1 year, and therefore was not included in our analyses. We further restricted these analyses to women with births in the previous 5 years to generate estimates of MNH services. Table 1 displays the DHS survey characteristics, final sample size, and number of clusters used in these analyses. We mapped cluster locations using ArcGIS software [31] and drew corresponding buffers of 2 km and 5 km around urban and rural locations (respectively) in order to minimize bias resulting from DHS displacement protocols, in accordance with DHS recommendations outlined by Burgert and colleagues [32].

**Table 1:** DHS survey used in study analysis and characteristics

Country	Citation	Survey Year	Survey Type	Clusters	Sample
Kenya	[19]	2003	Standard DHS-IV	399	2969
	[20]	2008/9	Standard DHS-V	397	3054
	[21]	2014	Standard DHS-VII	1585	11,151
Rwanda	[22]	2005	Standard DHS-V	462	4002
	[23]	2010	Standard DHS-VI	492	4746
	[24]	2014/15	Standard DHS-VII	492	4467
Tanzania	[25]	1999	Standard DHS-IV	176	1504
	[26]	2010	Standard DHS-VI	475	4019
	[27]	2015/16	Standard DHS-VII	608	5288
Uganda	[28]	2000/1	Standard DHS-IV	266	2790
	[29]	2006	Standard DHS-V	336	3420
	[30]	2011	Standard DHS-VI	400	3645

Finally, to allow for temporal analysis of model outcomes and spatial comparison, clusters at each survey year available were spatially linked to the most recent administrative II unit available for each country using ArcGIS software. Briefly, administrative units are subnational geographic areas used for administrative or political purposes, such as districts, regions, counties and states. In the United States, for example, administrative I units correspond to the state level, while administrative II units correspond to counties (with the exception of two states). Because the word for these geographic areas may vary substantially by country (ie, district, county, borough, etc.), the administrative II unit level used in these analyses is hereby referred to as the ‘district’ level for the purposes of this paper. We spatially linked survey data to the most current district boundaries available for each country, as both DHS and political boundaries in many of the study countries have changed since 1999, preventing direct comparison of change over time. Further, by disaggregating each country at a uniform district level, spatial comparisons across countries could be standardized.

### Absolute indices of temporal change

We employed a Bayesian inference framework using the Integrated Nested Laplace Approximation (INLA) package [33] in R [34] to spatially interpolate coverage estimates for ANC, SBA, and PNC at the district level throughout our study countries. Specifically, we used the Besag-York-Mollier (BYM-2) class of models within the INLA package, which have been shown to be particularly useful in mapping disease, as unstructured spatial effects can be added to account for region-specific variation [35, 36]. Our model was therefore defined as

$$ \mathrm{logit}\left({p}_{ij}\right)={\beta}_0+{\beta}_1{x}_{ij}+{\beta}_2{x}_{ij}\dots {\beta}_5{x}_{ij}+{f}_{spat}\left({admin}_j\right) $$

where logit(*p*_*ij*_) represents the odds of a woman’s most recent birth, *i,* in administrative unit, *j*, obtaining the corresponding health service (SBA, ANC, and PNC); *β*_0_ + *β*_1_*x*_*ij*_ + *β*_2_*x*_*ij*_…*β*_5_*x*_*ij*_ represents the fixed effects of the model as described below; and *f*_*spat*_(*admin*) represents the structured spatial effect of administrative unit, *j*, as a combination of both the structured and unstructured random effects, defined as

$$ {f}_{spat}\left({admin}_j\right)={f}_{struc}\left({admin}_j\right)+{f}_{unstruc}\left({admin}_j\right) $$

For these analyses, we assumed an uninformative prior distribution on model parameters to allow the data to drive model results, as no previous literature or data exist at this level for each year to inform our expectations of the spatial distribution of model outcomes. By assuming uninformative priors across all models at each time point available, this allowed for better comparison of model results. Similar approaches have been used previously [17] with adolescent health indicators using DHS data. The model outputs generated by this method represent a posterior distribution of possible estimates for each outcome at the district level. The mean of this distribution can therefore be taken to represent a point estimate for each geographic unit, while also allowing for reporting of standard distribution statistics (such as standard deviation and credibility intervals) which can be used to represent uncertainty surrounding each estimate. To visualize the absolute change over time among these indicators, we compared estimates for each country between the first and last surveys available.

Binary model outcomes included SBA, ANC, and PNC, while fixed model effects included urban/rural residence, education status, wealth quintile, maternal age, and parity, which have been defined in previous literature as important predictors of MNH services [15, 37, 38]. To maintain comparability across countries, we defined skilled birth attendance as births attended by a doctor, nurse, or auxiliary midwife for the most recent birth available. Antenatal care was defined amongst the most recent birth as 4+ antenatal care visits, while postnatal care was defined as a maternal check-up within 48 h of the most recent delivery by a health professional (doctor, nurse, or auxiliary midwife). For deliveries occurring at a health facility, we assumed postnatal care was provided by a health professional (as defined above) unless otherwise specified by the data. Lastly, we report model fit through the Deviance Information Criterion (DIC) values, which provide a measure of goodness-of-fit for Bayesian models, while adjusting for model complexity and effective number of parameters, with smaller DIC values representing better fitting models [39].

### Relative indices of temporal change

We examined relative indices of temporal change by quantifying the ratio between best-versus-worst modelled estimates among districts, with larger values representing increased gaps in coverage between districts, and smaller values nearing one representing decreasing spatial inequality. We further examined the temporal trend of spatial effects by reporting univariate logistic odds ratio (OR) using the ‘lme4’ package in R software [40] for each outcome and time point available. Similar approaches have been used by researchers at the DHS program [7] to temporally examine socioeconomic inequalities such as wealth in MNH. These analyses have reported coefficients for the richest quintiles as compared to the poorest, with values overlapping zero representing no statistically significant difference in services as predicted by wealth. We similarly report coefficients for DHS region with the best-performing (or highest coverage) for each outcome, as compared to the worst-performing (or lowest coverage) region, representing temporal trends in spatial inequalities divorced of modelled estimates. Specifically, we defined coverage as the proportion of women in the sample accessing a given service—for example, 90% of women reporting skilled attendance at birth would correspond to 90% coverage for this indicator. DHS regions used for reference and coefficients reported are outlined in Table A-1 (see Additional file 1: Appendix). More information on region boundaries used by the DHS can be found at spatialdata.dhsprogram.com/boundaries. In these analyses, values overlapping zero represent no significant effect of space in predicting odds of MNH outcome use, while increasing values represent a greater effect of space alone in predicting service utilisation.

### Validation

To validate model performance, we employed an out-of-sample validation technique, where 25% of the data were removed for validation purposes, while the remaining 75% were used for model training. We report standard validation statistics, including mean absolute error (MAE), mean square error (MSE), and pseudo-R2. Previous studies [16, 41] have employed similar statistics when interpolating surfaces using DHS data, and represent information on model precision, model bias, and variance explained, respectively.

## Results

### Absolute indices of temporal change

**Fig. 1:**
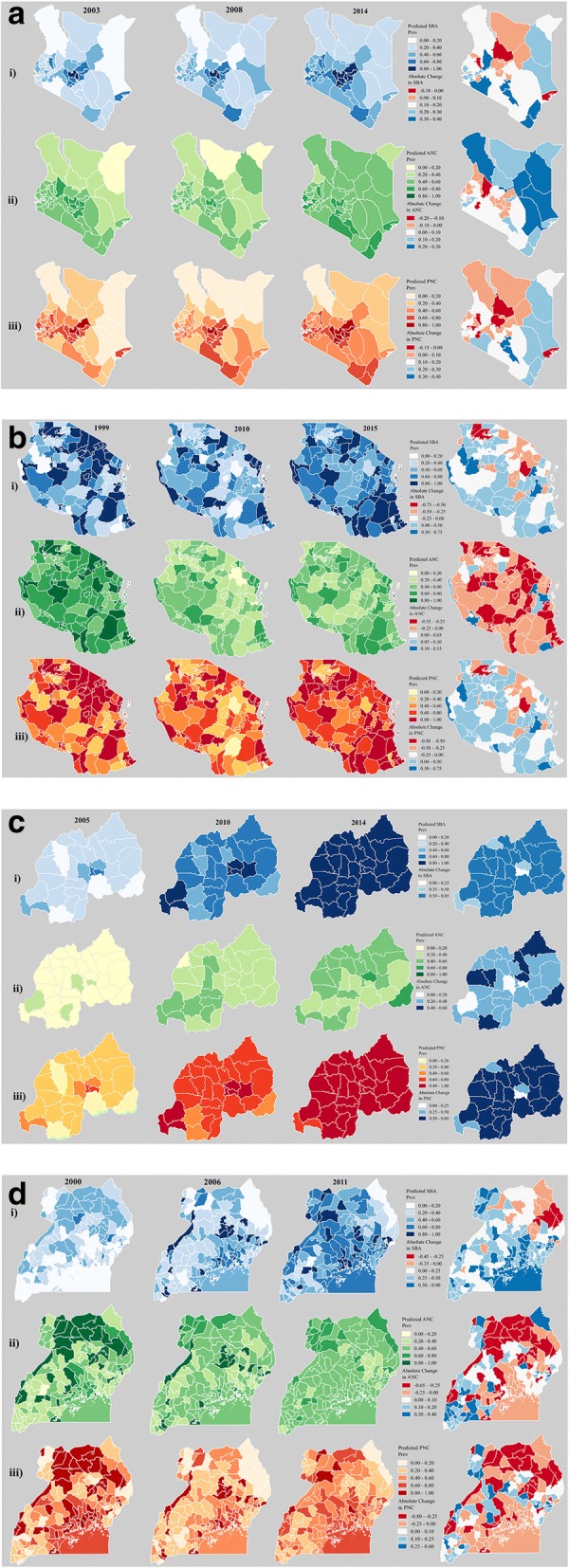
Predicted prevalence and absolute change in **i)** skilled birth attendance (blue), **ii)** 4+ antenatal care visits (green), and **iii)** postnatal check-up within 48 h (red) among administrative II units, among DHS data in **a)** Kenya, 2003–2014 **b)** Tanzania, 1999–2015, **c)** Rwanda, 2005–2014, and **d)** Uganda, 2000–2011

Figure 1 a-d show modelled prevalence estimates of skilled birth attendance (i), antenatal care (ii), and postnatal care (iii) at the district level, and corresponding absolute change over time in these estimates between the first and last time points available. For absolute change over time, areas in blue indicate an increasing prevalence estimates between the first and last time points, while areas in white represent a small, but increasing change over time, and areas in red indicate decreasing prevalence estimates over time. The corresponding absolute change estimates for each districts are labelled and ordered increasingly in Figure A.1a-d (see Additional file 1: Appendix), to facilitate visualisation of greatest to least improvement for each district

Rwanda (Fig. 1c) represented the only country with exclusively increasing prevalence over time amongst all three services, with as much as 85% increases in SBA for some districts and nearing universal coverage for both SBA and PNC. While more moderate gains were seen for antenatal care, every area within the country saw increases in prevalence, ranging to as high as a 60% increase. Equally encouraging, the greatest increases for Kenya (Fig. 1a) were primarily localized within the eastern region of Kenya, which has historically shown higher levels of wealth inequality and more adverse maternal health outcomes as compared to the rest of the country, particularly in the northern parts of the region [7]. Increases in this area were also seen amongst skilled birth attendance and postnatal care, however these were comparatively more conservative increases.

Conversely, Tanzania (Fig. 1b) had noticeable reductions in coverage throughout most of the country in utilisation of 4+ antenatal care visits, with some areas experiencing as much as a 55% reduction. This trend was predominantly driven by high coverage in ANC in 1999, which were substantially reduced in the 2010 and 2015 DHS surveys. This trend however, did not hold for skilled birth attendance and postnatal care, with much of the country experiencing improvement amongst these services. While Uganda (Fig. 1d) experienced improvement in skilled birth attendance over time, it experienced equally substantial decreases or little to no improvement in antenatal care and postnatal care in Northern and central Uganda. Similar to Tanzania, these trends were predominantly driven by high estimates during the 2000 DHS, decreasing with the 2006 and 2011 surveys.

**Fig. 2:**
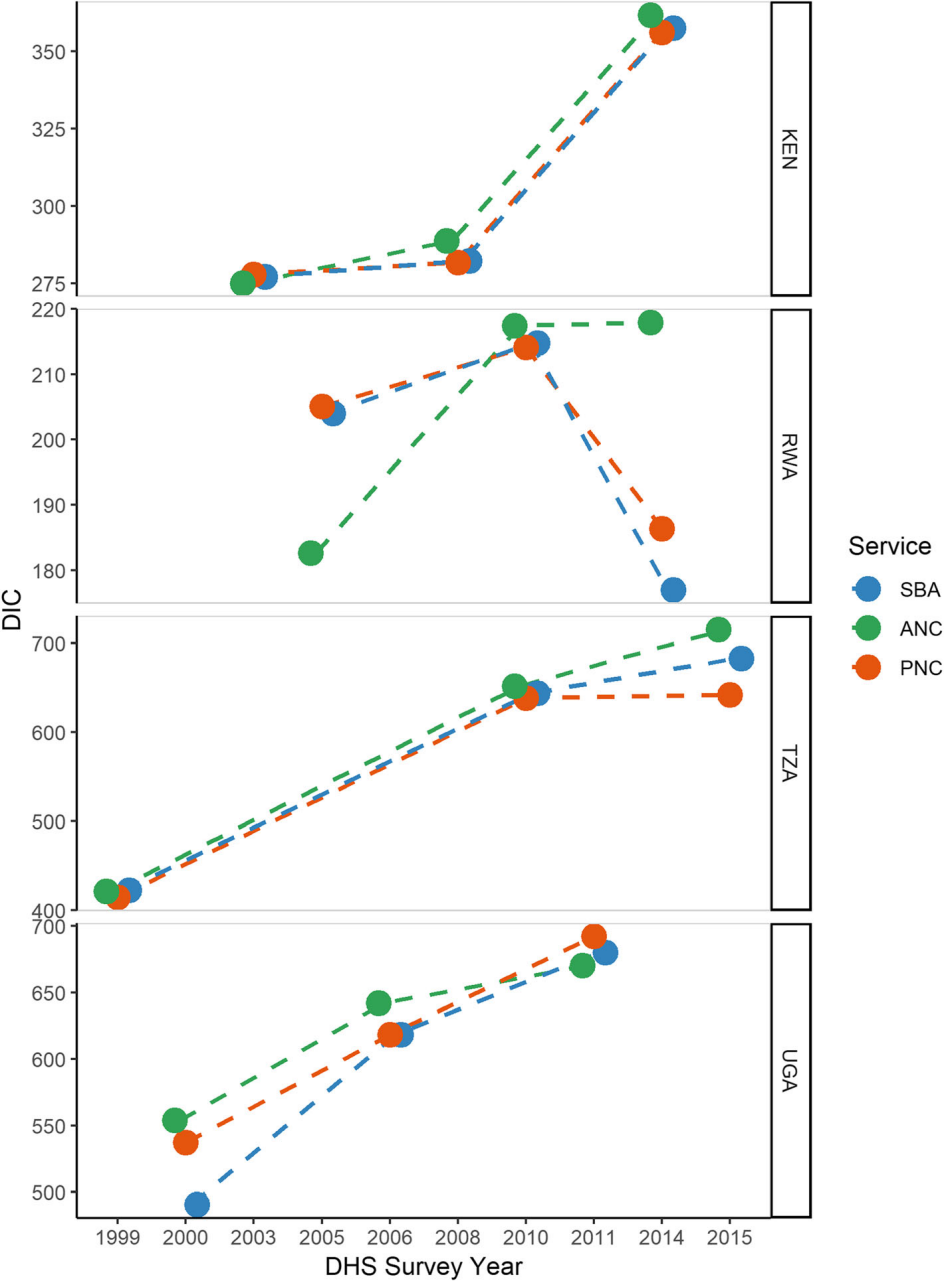
DIC values for skilled birth attendance (blue), 4+ antenatal care visits (green), and postnatal care (red) models, by country, DHS data, 1999–2015

Model outcomes, estimates and fit are outlined in Table A-2 (see Additional file 1: Appendix). Figure 2 compares model fits across outcomes by country, as measured by the DIC. Of note, the y-axis for DIC values varies by country, as these values should not be compared between countries. In general, SBA and PNC models tended to perform better as compared to ANC models, with the exception of the most recent models in Uganda, where models all performed similarly. DIC values also tended to increase over time, possibly due to increasing sample size (see Table 1), with the exception of Rwanda. Models in Rwanda tended to perform similarly in 2010, but had more variability in 2005 and 2014. However, this variability only ranged between 30 points, similar to models in other countries.

### Relative indices of temporal change

**Fig. 3:**
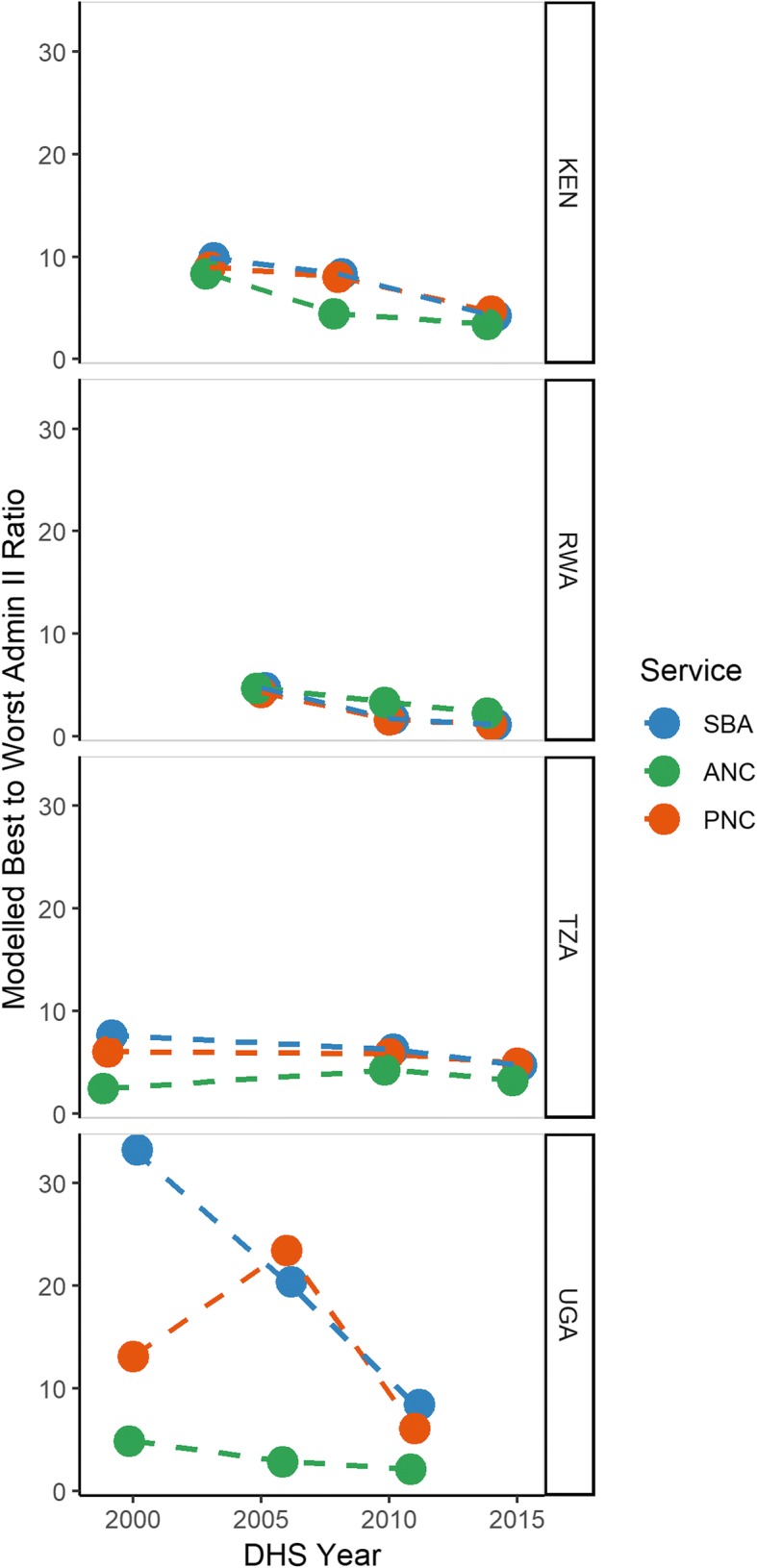
Ratio of modelled best-to-worst performing administrative II units over time for skilled birth attendance (blue), 4+ antenatal care visits (green), and postnatal care (red), by country, DHS data, 1999–2015

Figure 3 shows performance ratios of each outcome, reflecting the relative gap within countries between the district with the highest-versus-lowest prevalence estimates over time. Values closer to one therefore represent spatial homogeneity in coverage, while higher values represent more disparate gaps within countries amongst services. Across all countries, the lowest ratios tended to be among ANC, with the exception of Rwanda, while SBA and PNC tended to have greater ratios between the highest and lowest district estimates, suggesting greater spatial heterogeneity. Generally, ratios have typically decreased or remained stably low over time across all countries.

Historically, Rwanda had relatively small ratios which decreased over time, while Kenya had the highest inequalities in SBA, these were substantially reduced over time. Tanzania equally experienced reductions in SBA and PNC, but saw little to no change in ANC over time as it was already relatively low. Uganda also saw substantial improvement in reducing disparities over time for SBA and PNC (despite an increasing ratio for the year 2005), nearly halving the SBA ratio from 25 to 12 within the span of a decade. Regardless, the most recent ratios in Kenya and Tanzania across service utilisation still remained around 5, meaning the best performing region of the country had coverage about 5-times higher than the corresponding lowest region. Further, while Uganda saw improvement across services, ratios amongst SBA and PNC still represent the highest ratios across the region, suggesting there is still need for further improvement.

**Fig. 4:**
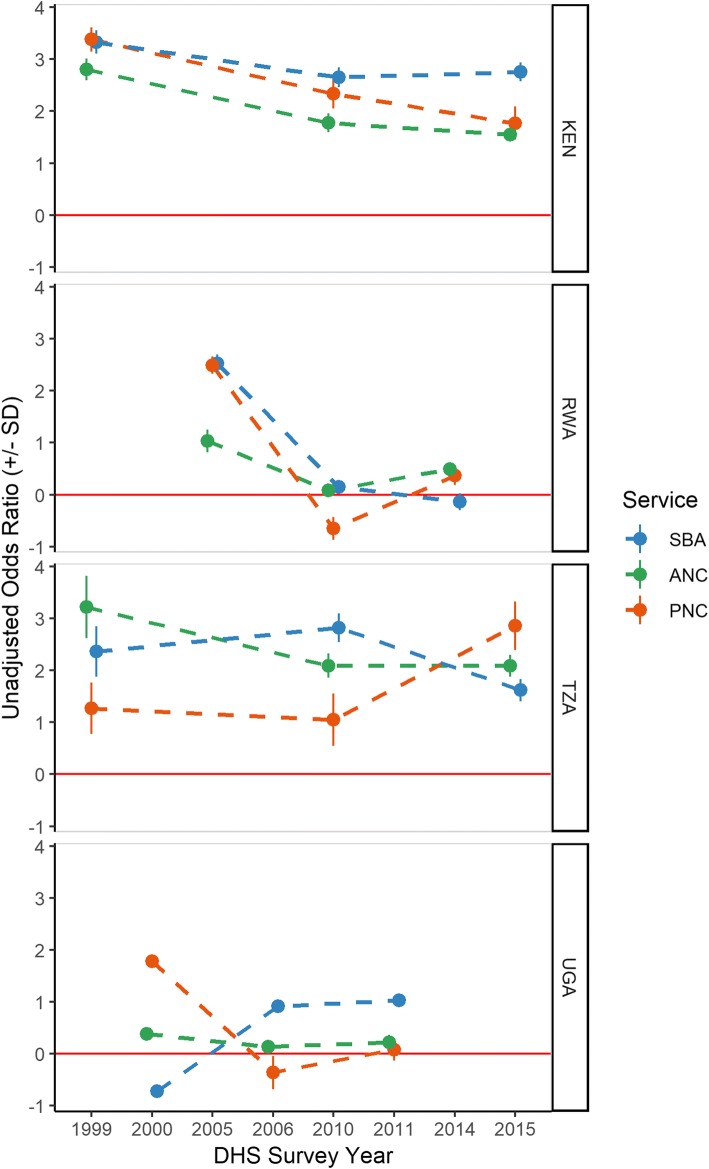
Unadjusted odds ratio of skilled birth attendance (blue), 4+ antenatal care visits (green), and postnatal care (red) over time, with worst performing DHS region as reference, DHS data, 1999–2015

Lastly, Fig. 4 shows results of the unadjusted logistic odds ratio, plotting coefficients of the DHS region with the highest corresponding coverage as compared to the region with the lowest coverage as reference. Positive, significant coefficients imply a statistically significant effect of space exists, while estimates spanning zero imply no significant difference across regions in likelihood of utilising SBA, ANC, or PNC. Historically, Rwanda saw high levels of inequality in utilising MNH services by space, but univariate logistic odds results suggest the effect of space has been substantially reduced over the decades, representing the country with the lowest odds ratios across services amongst the most recent survey. Of note, the odds of obtaining PNC in 2010 was negative, implying odds in the best performing regions were actually reduced as compared to the worst performing region. Kenya further saw decreasing ORs over time for both ANC and PNC, but saw little improvement for SBA, with Nairobi having 2 to 3 times higher odds of utilising skilled birth attendance over time as compared to the North Eastern region. Despite this improvement, Kenya’s ORs remain amongst the highest across the region, in combination with Tanzania.

In contrast to Rwanda and Kenya’s decreasing trends in ORs, Tanzania and Uganda had substantial variability in ORs over time. Encouragingly, the effect of space is relatively low in Uganda amongst ANC and PNC, but has increased over time for SBA. However, the odds of obtaining SBA amongst the DHS region with the highest SBA coverage versus the lowest was just over 1 in 2011, representing the lowest coefficient for SBA outside of Rwanda. Tanzania also saw an increasing effect of space over time for PNC, and further has high ORs along with Kenya.

### Model validation

Model validation results are show in Table 2. Generally, model fit was greatest for all services in Kenya, ranging from 88.4% variance explained for ANC in 2008 to as high as 99.7% in 2014 for SBA and PNC. Model fit was poorest for ANC in Uganda, with psudeo-R2 estimates of 0.138 and 0.239 for ANC in 2006 and 2014. In general, model fit was poorest for ANC for all years and countries, with the exception of Rwanda after 2010. Model precision and bias were predominantly uniform across countries, but was worst among Uganda models, potentially contributing to the low explained variance.

**Table 2:** Model validation results

	MSE	MAE	Psuedo -R^2^		MSE	MAE	Psuedo -R^2^
*Kenya*				*Tanzania*			
*2003*				*1999*			
SBA	0.0022	0.0344	0.949	SBA	0.0099	0.076	0.876
ANC	0.002	0.0337	0.925	ANC	0.0164	0.101	0.544
PNC	0.0019	0.0286	0.960	PNC	0.0098	0.0763	0.855
*2008*				*2010*			
SBA	0.0013	0.027	0.973	SBA	0.0049	0.0501	0.929
ANC	0.0021	0.0357	0.884	ANC	0.0067	0.0580	0.796
PNC	0.0017	0.030	0.967	PNC	0.0041	0.0455	0.938
*2014*				*2015*			
SBA	0.00009	0.0073	0.997	SBA	0.0043	0.0441	0.901
ANC	0.0001	0.0095	0.987	ANC	0.006	0.055	0.787
PNC	0.00008	0.0072	0.997	PNC	0.004	0.0376	0.925
*Rwanda*				*Uganda*			
*2005*				*2000*			
SBA	0.005	0.0538	0.80	SBA	0.024	0.115	0.656
ANC	0.003	0.0404	0.475	ANC	0.04	0.151	0.529
PNC	0.0043	0.0517	0.816	PNC	0.035	0.140	0.702
*2010*				*2006*			
SBA	0.002	0.033	0.887	SBA	0.030	0.127	0.657
ANC	0.001	0.0300	0.905	ANC	0.054	0.173	0.138
PNC	0.001	0.0326	0.877	PNC	0.028	0.124	0.684
*2014*				*2011*			
SBA	0.0008	0.024	0.704	SBA	0.025	0.119	0.678
ANC	0.0023	0.0365	0.795	ANC	0.050	0.171	0.239
PNC	0.0010	0.0270	0.738	PNC	0.033	0.136	0.534

## Discussion

The vast majority of maternal deaths can be prevented through routine health services, such as antenatal care and skilled birth attendance, or treated through timely interventions and prevention [42]. Within developing countries, however, the use of key life-saving interventions can be limited and inequitably distributed, and varies by country-specific contextual issues, such as funding and organization of health care or social and cultural issues [43]. Continuing MDG progress in preventing maternal deaths and achieving SDG targets of “ensuring healthy lives and promoting well-being for all at all ages” [6] will require more resolved spatial, temporal, and demographic information to identify and monitor persistent health inequalities. Examining temporal change in spatial inequalities of maternal health service use, however, requires reporting of both absolute and relative indices, as such measures often interact with each other synergistically and therefore require joint interpretation [5].

Here, we found that Rwanda was the only country to make substantial progress in both absolute and relative measures, increasing coverage amongst all services and reducing relative inequalities. Importantly, Rwanda had historically low relative inequality in service coverage (Fig. 3), suggesting that the increases in service coverage seen in Rwanda over the decades were experienced in spatially equitable manner, with most of the country improving together. These results are in line with previous findings, given that Rwanda was the only country in the region to achieve the MDG target 5a (reduce MMR by 75% between 1990 and 2015) [3]. After experiencing a devastating genocide in the mid-90s, Rwanda radically re-developed their health system, aimed at: 1) coordinating policy with external donors and government aid; 2) implementation of national-level health insurance; and, 3) introduction of a performance-based pay system for health workers [44]. This commitment to improving health across the country translated into a nearly 78% reduction in MMR throughout the MDG era, as well as a substantial reduction in under-5 mortality [9], and may contribute to the findings outlined in this study.

We found that Uganda has experienced both absolute and relative improvement over time in SBA across most of the country, while improvement in PNC and ANC lagged, particularly in the northern region. Previous studies [45] have similarly found poor outcomes in under-five mortality in this area, attributing the trend partly to the nearly two decades of armed conflict in the region which disrupted health systems and impacted the socio-economic stability across the region. Other studies [46] have found that while skilled attendance is high, other key MNH metrics such as vaccine coverage lag, and suggest that quality of care in Uganda is insufficient, resulting in delayed emergency treatment and insufficient supplies. Further studies [47] have found minimal increases over time in MNH indicators requiring multiple contacts with the health system, such as 4+ antenatal care visits, consistent with our findings. This suggests that while Uganda has rapidly increased the number and scale of maternal health interventions across the country, some areas (particularly in the northern regions) have historically and systematically lagged behind and require more deliberate efforts and focused interventions to further close the gap in MNH inequalities [45].

In Tanzania, we found a notable absolute decrease in coverage of 4+ antenatal care visits between 1999 and 2010, with little change through 2015. Regardless, northern and north-eastern Tanzania experienced a relative reduction over time across all services, suggesting the possibility that these areas are being left behind in improving access to or utilisation of maternal health services. Tanzania achieved MDG targets for child survival, and while previous studies [48] have noted that geographic inequalities in access to primary care at childbirth have been reduced, inequalities persist in actual hospital-based deliveries and antenatal care. Our findings are in line with these studies [49] suggesting maternal survival has lagged behind due to low coverage of maternal health services, with rural women bearing a disproportionate burden of risk. Examining potential bottlenecks to explain these patterns, Armstrong et al. [49] suggested that Tanzania must make progress in all four “benchmarks” of quality health systems, as the country experienced low density of health workers, poor availability of supplies at health facilities, and low levels of health financing, particularly in the Lake and Western districts. Despite this, we found ratios between districts generally decreased slightly, suggesting some progress is being made in reducing inequalities. Further, we found the odds of region alone in predicting SBA substantially declined and is remaining relatively stable with ANC, yet is noticeably increasing for PNC. These findings suggest that geographic inequalities in coverage of SBA are being reduced within country, but coverage in ANC and PNC must be followed closely and prioritized over the coming decades.

Finally, we found that Kenya experienced substantial improvement in absolute change of 4+ ANC visits over time, particularly with the eastern districts, but coverage in SBA and PNC decreased, particularly in the northern districts. We further found that while relative inequalities between districts are reducing across services, they still remain high compared to the rest of the region. Across the country, we also found that urban areas experienced substantially higher odds of obtaining MNH services (and particularly skilled birth attendance) as compared to rural areas, in line with previous research [7]. Nguhiu et al. similarly found that while overall coverage of MNH interventions has steadily increased over time and maternal inequities decreased within Kenya, coverage of individual interventions including antenatal care and skilled birth attendance remained stubbornly low and inequitable, with ANC experiencing the most inequitable coverage [2]. They suggest that increased overall coverage may be linked to increasing per capita health expenditure within the country, as well as urbanization and expansion of lower level health facilities over the decades, but propose that interventions directed at those services lagging behind should be accordingly prioritised over the coming decades.

### Limitations

As this work primarily utilises estimates generated using statistical inference, it is subject to several limitations, including survey and sampling errors inherent to DHS data. Particular to spatial interpolation at levels below the DHS region, these data were collected using methods representative at geographic units which are different from the measures we report, potentially resulting in model uncertainty and errors. However, the DHS program endorses use of geospatial interpolation methods such as Bayesian inference, particularly because of the ability to quantify this uncertainty in posterior distribution estimates [16]. The DHS data is also subject to temporal biases, as it is collected at varying intervals between countries, and information on maternal services are collected for up to 5 years previous. This represents a potential time lag in temporal analyses, and the trends reported here may not represent the current picture of MNH service utilisation in these countries. Further, with some of the countries used in these analyses, the most recent DHS available is upwards of 5 years old, representing an important avenue for future analyses to continue to examine these trends using more recent data. Lastly, the actual hospital or health facility used for the services used was not included in these analyses, as the DHS does not report this information. This could potentially bias model results, as the hospital or health facility may be outside of the respondent’s surveyed district. Actual health facility used should be included in future research if available, as well as cross-border movement.

While the scope of this paper is to report temporal trends in spatial inequalities of MNH service use over time, future work should aim to explain why these patterns are occurring to potentially offer insight into policy interventions aimed at maintaining progress to ensure no populations are left behind in accessing MNH services. Future research should examine factors at several socio-ecological levels, including the individual, cultural, and national level, as well as quality of care provided. Spatial context such as travel time to health facilities and actual health facilities used should also be taken into account [50].

## Conclusions

This study is the first to report model-based estimates at the district level for several time points across the EAC region, as well as report temporal trends in both absolute and relative measures of spatial inequalities. We found that relative inequality between districts have generally decreased or remained stably low over time across all countries, suggesting improvements are being made to reduce the gap between the geographic areas with the highest and lowest coverage in services. We further found that Rwanda in particular was successful in reducing relative inequalities over time, as well as increasing absolute coverage across all MNH services. Despite this progress, we found that relative measures of spatial inequalities across the region indicate that the effect of space is becoming more prominent over the decades among PNC in particular, suggesting a worrying trend that should be monitored closely throughout the SDG era and examined further with new sources of DHS data or other household surveys.

These analyses demonstrate how the use of spatial and temporal disaggregation methods can be used to monitor the evolution of health inequalities over the SDG period. Our results highlight the need for continued disaggregation of these data over time, which will be key in improving health amongst all populations across the East African Community. Lastly, in-country uptake and adoption of GIS-enabled analytics facilitating concurrent temporal and spatial analysis of socio-economic, environmental and health systems level determinants will be key to development of precise policy actions for addressing areas with intractable health challenges and ensuring that SDG health targets are met by 2030.

## Additional file

Additional file 1: Appendix. Supplementary information containing 1) unadjusted logistic regression results; 2) model fit, mean posterior estimates and hyperparameters; and 3) ordered absolute change in indicators by administrative II unit. (DOCX 1505 kb)

## Abbreviations

ANC: Antenatal care
DHS: Demographic and Health Surveys
DIC: Deviance Information Criterion
EAC: East African Community
GPS: Global position system
INLA: Integrated Nested Laplace Approximation
MDG: Millennium Development Goal
MMR: Maternal mortality ratio
MNH: Maternal and newborn health
OR: Odds ratio
PNC: Postnatal care
SBA: Skilled birth attendance
SDG: Sustainable Development Goal
UN: United Nations

## References

[1] Boerma J, Bryce J, Kinfu Y, Axelson H, Victora C (2008). Mind the gap: equity and trends in coverage of maternal, newborn, and child health services in 54 countdown countries. Lancet Lond Engl.

[2] Nguhiu PK, Barasa EW, Chuma J (2017). Determining the effective coverage of maternal and child health services in Kenya, using demographic and health survey data sets: tracking progress towards universal health coverage. Tropical Med Int Health.

[3] WHO, UNICEF, World Bank, UNFPA, United Nations population division. Trends in maternal mortality: 1990 to 2015. World Health Organization; 2015. http://apps.who.int/iris/bitstream/10665/194254/1/9789241565141_eng.pdf?ua=1. Accessed 20 Jan 2017.

[4] WHO. State of inequality: Reproductive, maternal, newborn and child health. Geneva, Switzerland: World Health Organization; 2015. http://apps.who.int/iris/bitstream/10665/164590/1/9789241564908_eng.pdf?ua=1&ua=1. Accessed 16 Feb 2017.

[5] Barros AJD, Victora CG (2013). Measuring coverage in MNCH: determining and interpreting inequalities in coverage of maternal, newborn, and child health interventions. PLoS Med.

[6] WHO. Health in 2015: From MDGs, millennium development goals to SDGs, sustainable development goals. Geneva, Switzerland: World Health Organization; 2015. http://www.who.int/gho/publications/mdgs-sdgs/en/. Accessed 19 Apr 2018.

[7] Assaf S, Pullum T. Levels and Trends in Maternal and Child Health Disparities by Wealth and Region in Eleven Countries with DHS Surveys. Rockville, Maryland, USA: ICF International; 2016. https://www.dhsprogram.com/pubs/pdf/CR42/CR42.pdf. Accessed 13 Mar 2017.

[8] Kerber KJ, de Graft-Johnson JE, Bhutta ZA, Okong P, Starrs A, Lawn JE (2007). Continuum of care for maternal, newborn, and child health: from slogan to service delivery. Lancet.

[9] Golding N, Burstein R, Longbottom J, Browne AJ, Fullman N, Osgood-Zimmerman A (2017). Mapping under-5 and neonatal mortality in Africa, 2000–15: a baseline analysis for the sustainable development goals. Lancet.

[10] Graetz N, Friedman J, Osgood-Zimmerman A, Burstein R, Biehl MH, Shields C (2018). Mapping local variation in educational attainment across Africa. Nature.

[11] Osgood-Zimmerman A, Millear AI, Stubbs RW, Shields C, Pickering BV, Earl L (2018). Mapping child growth failure in Africa between 2000 and 2015. Nature.

[12] Victora CG, Requejo JH, Barros AJD, Berman P, Bhutta Z, Boerma T (2016). Countdown to 2015: a decade of tracking progress for maternal, newborn, and child survival. Lancet.

[13] Alkema L, Chou D, Hogan D, Zhang S, Moller A-B, Gemmill A (2016). Global, regional, and national levels and trends in maternal mortality between 1990 and 2015, with scenario-based projections to 2030: a systematic analysis by the UN maternal mortality estimation inter-agency group. Lancet.

[14] Black RE, Cousens S, Johnson HL, Lawn JE, Rudan I, Bassani DG (2010). Global, regional, and national causes of child mortality in 2008: a systematic analysis. Lancet.

[15] Ruktanonchai CW, Ruktanonchai NW, Nove A, Lopes S, Pezzulo C, Bosco C (2016). Equality in maternal and newborn health: modelling geographic disparities in utilisation of Care in Five East African Countries. PLoS One.

[16] Gething P, Tatem A, Bird T, Burgert C. Creating spatial interpolation surfaces with DHS data. Rockville, Maryland, USA: ICF international; 2015. http://dhsprogram.com/pubs/pdf/SAR11/SAR11.pdf. Accessed 28 Mar 2017.

[17] Neal S, Ruktanochai CW, Chandra-Mouli V, Harvey C, Matthews Z, Raina N, et al. Using geospatial modelling to estimate the prevalence of adolescent first births in Nepal. BMJ Glob Health. 2018. 10.1136/bmjgh-2018-000763. 10.1136/bmjgh-2018-000763 PMC6606082 31321088

[18] SAS Institute Inc. SAS version 9.4. Cary, NC, USA: SAS Institute Inc.; 2013.

[19] Central Bureau of Statistics - CBS/Kenya, Ministry of Health - MOH/Kenya, ORC Macro. Kenya Demographic and Health Survey 2003. Calverton, Maryland, USA: CBS, MOH, and ORC Macro; 2004. http://dhsprogram.com/pubs/pdf/FR151/FR151.pdf. Accessed 29 Mar 2018.

[20] Kenya National Bureau of Statistics - KNBS, National AIDS Control Council/Kenya, National AIDS/STD Control Programme/Kenya, Ministry of Public Health and Sanitation/Kenya, Kenya Medical Research Institute. Kenya Demographic and Health Survey 2008–09. Calverton, Maryland, USA: KNBS and ICF Macro; 2010. http://dhsprogram.com/pubs/pdf/FR229/FR229.pdf. Accessed 29 Mar 2018.

[21] Kenya National Bureau of Statistics, Ministry of Health/Kenya, National AIDS Control Council/Kenya, Kenya Medical Research Institute, National Council for Population and Development/Kenya. Kenya Demographic and Health Survey 2014. Rockville, MD, USA; 2015. http://dhsprogram.com/pubs/pdf/FR308/FR308.pdf. Accessed 29 Mar 2018.

[22] Institut National de la Statistique du Rwanda - INSR, ORC Macro. Rwanda Demographic and Health Survey 2005. Calverton, Maryland, USA: INSR and ORC Macro; 2006. http://dhsprogram.com/pubs/pdf/FR183/FR183.pdf. Accessed 29 Mar 2018.

[23] National Institute of Statistics of Rwanda - NISR, Ministry of Health - MOH/Rwanda, ICF International. Rwanda Demographic and Health Survey 2010. Calverton, Maryland, USA: NISR/Rwanda, MOH/Rwanda, and ICF International; 2012. http://dhsprogram.com/pubs/pdf/FR259/FR259.pdf. Accessed 29 Mar 2018.

[24] National Institute of Statistics of Rwanda, Ministry of Finance and Economic Planning/Rwanda, Ministry of Health/Rwanda, ICF International. Rwanda Demographic and Health Survey 2014–15. Kigali, Rwanda: National Institute of Statistics of Rwanda, Ministry of Finance and Economic Planning/Rwanda, Ministry of Health/Rwanda, and ICF International; 2016. http://dhsprogram.com/pubs/pdf/FR316/FR316.pdf. Accessed 29 Mar 2018.

[25] National Bureau of Statistics/Tanzania, Macro International. Tanzania Reproductive and Child Health Survey 1999. Calverton, Maryland, USA: National Bureau of Statistics/Tanzania and Macro International; 2000. http://dhsprogram.com/pubs/pdf/FR112/FR112.pdf. Accessed 29 Mar 2018.

[26] National Bureau of Statistics - NBS/Tanzania, ICF Macro. Tanzania Demographic and Health Survey 2010. Dar es Salaam, Tanzania: NBS/Tanzania and ICF Macro; 2011. https://www.dhsprogram.com/pubs/pdf/FR243/FR243. Accessed 29 Mar 2018.

[27] Ministry of Health CD, Ministry of Health - MoH/Zanzibar, National Bureau of Statistics - NBS/Tanzania, Office of Chief Government Statistician - OCGS/Zanzibar, ICF. Tanzania Demographic and Health Survey and Malaria Indicator Survey 2015-2016. Dar es Salaam, Tanzania: MoHCDGEC, MoH, NBS, OCGS, and ICF; 2016. http://dhsprogram.com/pubs/pdf/FR321/FR321.pdf. Accessed 29 Mar 2018.

[28] Uganda Bureau of Statistics - UBOS, ORC Macro. Uganda Demographic and Health Survey 2000-2001. Calverton, Maryland, USA: UBOS and ORC Macro; 2001. http://dhsprogram.com/pubs/pdf/FR128/FR128.pdf. Accessed 29 Mar 2018.

[29] Uganda Bureau of Statistics - UBOS, Macro International. Uganda Demographic and Health Survey 2006. Calverton, Maryland, USA: UBOS and Macro International; 2007. http://dhsprogram.com/pubs/pdf/FR194/FR194.pdf. Accessed 29 Mar 2018.

[30] Uganda Bureau of Statistics - UBOS, ICF International. Uganda Demographic and Health Survey 2011. Kampala: UBOS and ICF International; 2012. http://dhsprogram.com/pubs/pdf/FR194/FR194.pdf. Accessed 29 Mar 2018.

[31] Environmental Syst Res Institute. ArcGIS Desktop: Release 10.2.2. Redlands; 2014.

[32] Burgert CR, Colston J, Roy T, Zachary B. Geographic displacement procedure and georeferenced data release policy for the demographic and health surveys. Calverton, Maryland, USA: ICF international; 2013. http://dhsprogram.com/pubs/pdf/SAR7/SAR7.pdf. Accessed 29 Mar 2018.

[33] Rue H, Martino S, Chopin N (2009). Approximate Bayesian inference for latent Gaussian models by using integrated nested Laplace approximations. J R Stat Soc Ser B Stat Methodol.

[34] R Development Core Team. R: A Language and Environment for statistical computing. Vienna, Austria: R Foundation for Statistical Computing; 2017. http://www.R-project.org.

[35] Niragire F, Achia TNO, Lyambabaje A, Ntaganira J (2015). Bayesian mapping of HIV infection among women of reproductive age in Rwanda. PLoS One.

[36] Riebler A, Sørbye SH, Simpson D, Rue H. An intuitive Bayesian spatial model for disease mapping that accounts for scaling. ArXiv160101180 Stat 2016. http://arxiv.org/abs/1601.01180. Accessed 16 Jan 2018. 10.1177/0962280216660421 27566770

[37] Neal SE, Chandra-Mouli V, Chou D (2015). Adolescent first births in East Africa: disaggregating characteristics, trends and determinants. Reprod Health.

[38] Gabrysch S, Campbell OM (2009). Still too far to walk: literature review of the determinants of delivery service use. BMC Pregnancy Childbirth.

[39] Spiegelhalter DJ, Best NG, Carlin BP, Van Der Linde A (2002). Bayesian measures of model complexity and fit. J R Stat Soc Ser B Stat Methodol.

[40] Bates D, Mächler M, Bolker B, Walker S (2014). Fitting linear mixed-effects models using lme4. ArXiv14065823 Stat.

[41] Bosco C, Alegana V, Bird T, Pezzulo C, Bengtsson L, Sorichetta A (2017). Exploring the high-resolution mapping of gender-disaggregated development indicators. J R Soc Interface.

[42] Bailey P, Paxton A, Lobis S, Fry D (2006). The availability of life-saving obstetric services in developing countries: an in-depth look at the signal functions for emergency obstetric care. Int J Gynecol Obstet.

[43] Say L, Raine R (2007). A systematic review of inequalities in the use of maternal health care in developing countries: examining the scale of the problem and the importance of context. Bull World Health Organ.

[44] Logie DE, Rowson M, Ndagije F (2008). Innovations in Rwanda’s health system: looking to the future. Lancet.

[45] Ayiko R, Antai D, Kulane A (2009). Trends and determinants of under-five mortality in Uganda. East Afr J Public Health.

[46] Parkhurst JO, Penn-Kekana L, Blaauw D, Balabanova D, Danishevski K, Rahman SA (2005). Health systems factors influencing maternal health services: a four-country comparison. Health Policy.

[47] Roberts DA, Ng M, Ikilezi G, Gasasira A, Dwyer-Lindgren L, Fullman N (2015). Benchmarking health system performance across regions in Uganda: a systematic analysis of levels and trends in key maternal and child health interventions, 1990–2011. BMC Med.

[48] Hanson C, Gabrysch S, Mbaruku G, Cox J, Mkumbo E, Manzi F (2017). Access to maternal health services: geographical inequalities, United Republic of Tanzania. Bull World Health Organ.

[49] Armstrong CE, Martínez-Álvarez M, Singh NS, John T, Afnan-Holmes H, Grundy C (2016). Subnational variation for care at birth in Tanzania: is this explained by place, people, money or drugs?. BMC Public Health.

[50] Makanga PT, Schuurman N, von Dadelszen P, Firoz T (2016). A scoping review of geographic information systems in maternal health. Int J Gynecol Obstet.

