# Supplementary material for: Temporal trends in spatial inequalities of maternal and newborn health services among four east African countries, 1999–2015
Source: BMC Public Health. 2018 Dec 4;18:1339. doi: 10.1186/s12889-018-6241-8 (PMC6278077; doi:10.1186/s12889-018-6241-8)

**APPENDIX**

| **Table A-1**. Unadjusted logistic regression results, with worst performing DHS region as reference, as compared to best performing DHS region | | | | |
| --- | --- | --- | --- | --- |
| **Outcome** | **Year** | **Worst performing Region (Reference)** | **Best performing region**  **(Coefficient)** | **Model**  **coefficient**  **(±SD)** |
| ***Kenya*** | | | | |
| SBA | 2003 | North Eastern | Nairobi | 3.33 (0.22) |
| ANC |  | North Eastern | Nairobi | 2.80 (0.21) |
| PNC |  | North Eastern | Nairobi | 3.38 (0.23) |
| SBA | 2008 | Western | Nairobi | 2.65 (0.19) |
| ANC |  | North Eastern | Nairobi | 1.78 (0.18) |
| PNC |  | North Eastern | Nairobi | 2.34 (0.29) |
| SBA | 2014 | North Eastern | Nairobi | 2.76 (0.18) |
| ANC |  | North Eastern | Nairobi | 1.55 (0.13) |
| PNC |  | North Eastern | Central | 1.77 (0.32) |
| **Outcome** | **Year** | **Worst performing Region (Reference)** | **Best performing region** | **Model**  **coefficient**  **(±SD)** |
| ***Tanzania*** | | | | |
| SBA | 1999 | Kigoma | Dar Es Salaam | 2.37 (0.49) |
| ANC |  | Kigoma | Dar Es Salaam | 3.22 (0.6) |
| PNC |  | Kigoma | Dar Es Salaam | 1.27 (0.5) |
| SBA | 2010 | Pemba North | Dar Es Salaam | 2.82 (0.27) |
| ANC |  | Pemba North | Dar Es Salaam | 2.09 (0.23) |
| PNC |  | Mwanza | Ruvuma | 1.05 (0.51) |
| SBA | 2015 | Pemba South | Dar Es Salaam | 1.62 (0.21) |
| ANC |  | Pemba North | Dar Es Salaam | 2.09 (0.21) |
| PNC |  | Pemba South | Dar Es Salaam | 2.85 (0.47) |

| **Outcome** | **Year** | **Worst performing Region (Reference)** | **Best performing region** | **Model**  **coefficient**  **(±SD)** |
| --- | --- | --- | --- | --- |
| ***Rwanda*** | | | | |
| SBA | 2005 | Gikongoro | Kigali | 2.53 (0.17) |
| ANC |  | Kigali Ngali | Cyangugu | 1.03 (0.22) |
| PNC |  | Gikongoro | Kigali | 2.49 (0.17) |
| SBA | 2010 | North | South | 0.15 (0.08) |
| ANC |  | Kigali | South | 0.08 (0.09) |
| PNC |  | Kigali | South | -0.65 (0.22) |
| SBA | 2014 | North | South | -0.13 (0.16) |
| ANC |  | Kigali | South | 0.49 (0.09) |
| PNC |  | Kigali | South | 0.37 (0.18) |
| **Outcome** | **Year** | **Worst performing Region (Reference)** | **Best performing region** | **Model**  **coefficient**  **(±SD)** |
| ***Uganda*** | | | | |
| SBA | 2000 | Northern | Central | -0.72 (0.12) |
| ANC |  | Northern | Central | 0.38 (0.11) |
| PNC |  | Northern | Central | 1.78 (0.12) |
| SBA | 2006 | West Nile | East Central | 0.91 (0.14) |
| ANC |  | North | Central 1 | 0.14 (0.13) |
| PNC |  | West Nile | East Central | -0.36 (0.32) |
| SBA | 2011 | Southwest | East Central | 1.03 (0.13) |
| ANC |  | Central 1 | Karamoja | 0.23 (0.14) |
| PNC |  | Southwest | East Central | 0.08 (0.21) |

| **Table A-2** Model fit, mean posterior estimates and hyperparameters of modelled MNH outcomes | | | | | |
| --- | --- | --- | --- | --- | --- |
| **Fixed Effects** | **SBA** | | **ANC** | | **PNC** |
|  | *Mean Posterior Estimate (SD)* | | | | |
| ***Kenya*** | | | | | |
| *2003* | | | | | |
| Intercept  Rural/Urban  Education  Wealth  Age  Births  DIC  MLL | 7.39 (2.33)  -0.04 (0.45)  -1.02 (0.44)  -1.29 (0.57)  -0.13 (0.07)  -2.01 (0.7)  277.06  -141.21 | | -1.83 (2.01)  -0.12 (0.39)  -1.1 (0.42)  -0.45 (0.5)  0.07 (0.06)  0.24 (0.63)  275.07  -139.50 | | 4.49 (2.57)  0.23 (0.5)  -1.4 (0.48)  -1.4 (0.62)  -0.07 (0.08)  -1.25 (0.77)  277.76  -145.49 |
| *2008* | | | | | |
| Intercept  Rural/Urban  Education  Wealth  Age  Births  DIC  MLL | 5.14 (2.53)  -0.51 (0.65)  -0.32 (0.62)  -1.96 (0.7)  -0.05 (0.08)  -1.59 (0.93)  282.26  -148.14 | | 0.18 (1.93)  -0.52 (0.46)  -0.34 (0.42)  -0.85 (0.54)  0.03 (0.06)  -0.19 (0.65)  288.76  -144.44 | | 4.47 (2.29)  -0.48 (0.58)  -0.86 (0.55)  -1.82 (0.63)  -0.04 (0.07)  -1.44 (0.85)  281.75  -143.96 |
| *2014* | | | | | |
| Intercept  Rural/Urban  Education  Wealth  Age  Births  DIC  MLL | 0.95 (3.7)  -0.16 (0.7)  -0.67 (0.58)  -2.26 (0.78)  0.09 (0.12)  -1.16 (1.09)  357.55  -205.56 | | 3.61 (2.56)  -0.55 (0.46)  -0.22 (0.42)  -0.43 (0.51)  -0.09 (0.08)  -0.11 (0.72)  361.77  -197.33 | | 0.92 (3.83)  -0.14 (0.72)  -0.91 (0.61)  -2.29 (0.81)  0.08 (0.13)  -0.98 (1.14)  356.08  -205.71 |
| **Fixed Effects** | **SBA** | | **ANC** | | **PNC** |
|  | *Mean Posterior Estimate (SD)* | | | | |
| ***Tanzania*** | | | | | |
| *1999* | | | | | |
| Intercept  Rural/Urban  Education  Wealth  Age  Births  DIC  MLL | 3.97 (1.48)  -0.96 (0.32)  -1.48 (0.54)  -1.05 (0.42)  -0.02 (0.05)  -1.27 (0.41)  422.80  -163.80 | | 2.18 (1.13)  0.01 (0.24)  -0.56 (0.4)  -0.37 (0.33)  -0.001 (0.04)  -0.83 (0.32)  422.09  -151.68 | | 4.25 (1.39)  -0.61 (0.31)  -1.55 (0.5)  -1.21 (0.4)  -0.01 (0.05)  -1.47 (0.39)  414.53  -156.03 |
| *2010* | | | | | |
| Intercept  Rural/Urban  Education  Wealth  Age  Births  DIC  MLL | 6.94 (1.31)  -0.87 (0.34)  -1.81 (0.56)  -1 (0.44)  -0.08 (0.04)  -1.92 (0.38)  644.04  -297.88 | | 1.44 (0.9)  -0.43 (0.23)  -0.27 (0.39)  -0.5 (0.31)  0.04 (0.03)  -1.48 (0.27)  652.56  -280.60 | | 7.55 (1.39)  -0.93 (0.36)  -2.04 (0.59)  -0.93 (0.46)  -0.1 (0.04)  -1.74 (0.4)  638.43  -299.45 |
| *2015* | | | | | |
| Intercept  Rural/Urban  Education  Wealth  Age  Births  DIC  MLL | 4.41 (1.21)  -0.46 (0.28)  -2.52 (0.56)  -0.72 (0.35)  0 (0.04)  -1.69 (0.35)  682.95  -312.88 | | 2.07 (0.95)  -0.24 (0.21)  0 (0.44)  -0.53 (0.28)  0.01 (0.03)  -1.29 (0.28)  715.99  -318.71 | | 7.25 (1.47)  -0.68 (0.34)  -3.3 (0.66)  -0.64 (0.42)  -0.03 (0.05)  -2.59 (0.42)  641.95  -298.86 |
| ***Rwanda*** | | | | | |
| *2005* | | | | | |
| Intercept  Rural/Urban  Education  Wealth  Age  Births  DIC  MLL | 1.32 (2.15)  -1.43 (0.34)  -1.9 (0.92)  0 (31.62)  0.02 (0.05)  -0.63 (0.75)  169.60  -79.95 | | -5.84 (2.68)  -0.29 (0.4)  -2.92 (1.17)  0 (31.62)  0.05 (0.07)  2.13 (0.91)  144.47  -65.33 | | 0.32 (2.21)  -1.57 (0.35)  -1.57 (0.94)  0 (31.62)  0.05 (0.05)  -0.59 (0.76)  170.54  -80.75 |
| *2010* | | | | | |
| Intercept  Rural/Urban  Education  Wealth  Age  Births  DIC  MLL | 0.96 (3.07)  -0.7 (0.63)  -1.66 (1.19)  -0.98 (0.95)  0.03 (0.08)  0.05 (1.01)  181.69  -93.78 | | -3.4 (3.39)  -1.22 (0.66)  -1.05 (1.33)  1.76 (0.99)  0.14 (0.09)  -0.69 (1.1)  182.98  -96.71 | | 2.94 (3.06)  -0.03 (0.63)  -2.02 (1.18)  -1.24 (0.96)  -0.01 (0.08)  -0.77 (1.01)  180.2  -92.80 |
| *2014* | | | | | |
| Intercept  Rural/Urban  Education  Wealth  Age  Births  DIC  MLL | -0.82 (4.49)  -0.67 (1)  -4.75 (2.42)  -0.33 (1.45)  0.11 (0.13)  1.11 (1.85)  137.26  -64.22 | | -3.64 (2.66)  -0.45 (0.57)  -2.73 (1.42)  1.48 (0.89)  0.05 (0.07)  1.52 (1.06)  179.75  -90.14 | | 0.84 (3.97)  1.11 (0.87)  -1.18 (2.15)  -1.57 (1.33)  0.06 (0.11)  -0.31 (1.6)  148.89  -72.30 |
| **Fixed Effects** | **SBA** | | **ANC** | | **PNC** |
|  | *Mean Posterior Estimate (SD)* | | | | |
| ***Uganda*** | | | | | |
| *2000* | | | | | |
| Intercept  Rural/Urban  Education  Wealth  Age  Births  DIC  MLL | -0.7 (1.49)  -1.18 (0.37)  -0.91 (0.67)  0.48 (0.3)  -0.02 (0.05)  0.48 (0.44)  355.28  -115.51 | | 0.91 (1.11)  -0.87 (0.29)  -0.61 (0.49)  -0.15 (0.23)  0 (0.03)  -0.13 (0.33)  394.81  -129.15 | | 4.61 (1.28)  -1.72 (0.34)  -1.25 (0.55)  0.07 (0.25)  -0.08 (0.04)  -0.47 (0.37)  374.86  -121.04 |
| *2006* | | | | | |
| Intercept  Rural/Urban  Education  Wealth  Age  Births  DIC  MLL | 2.59 (1.17)  -1.39 (0.53)  -0.99 (0.54)  -1.07 (0.42)  0.01 (0.04)  -0.81 (0.37)  420.75  -142.35 | | 0.54 (0.88)  -0.6 (0.32)  -0.06 (0.38)  -0.07 (0.29)  0.02 (0.03)  -0.43 (0.3)  444.63  -144.13 | | 2.55 (1.19)  -1.01 (0.54)  -0.96 (0.56)  -1.3 (0.44)  0.02 (0.04)  -0.99 (0.38)  417.22  -142.13 |
| *2011* | | | | | |
| Intercept  Rural/Urban  Education  Wealth  Age  Births  DIC  MLL | 1.86 (1.18)  -1.47 (0.4)  -0.85 (0.52)  -1.39 (0.49)  0.04 (0.03)  -0.36 (0.35)  453.70  -161.03 | | 0.46 (0.93)  -0.45 (0.29)  0.96 (0.4)  -0.54 (0.36)  -0.01 (0.03)  0.1 (0.3)  483.55  -166.45 | | 1.63 (1.06)  -0.55 (0.34)  -0.43 (0.48)  -1.26 (0.43)  0 (0.03)  -0.4 (0.33)  476.54  -169.32 |
| **Hyperparameter**  **(Spatial Precision)** | **SBA** | | **ANC** | | **PNC** |
|  | *Mean Posterior Estimate (SD)* | | | | |
| ***Kenya*** | | | | | |
| 2003 | 5.1 (1.8) | 6.2 (2.2) | | 3.6 (1.1) | |
| 2008 | 3.5 (1.1) | 8.0 (3.2) | | 4.8 (1.7) | |
| 2014 | 6.9 (6.4) | 14.1 (9.2) | | 6.9 (4.9) | |
| ***Tanzania*** | | | | | |
| 1999 | 2.0 (0.6) | 5.8 (2.6) | | 2.5 (0.8) | |
| 2010 | 2.0 (0.4) | 5.6 (1.5) | | 1.8 (0.4) | |
| 2015 | 3.1 (0.6) | 5.6 (1.3) | | 2.1 (0.4) | |
| ***Rwanda*** | | | | | |
| 2005 | 36.7 (53.5) | 111.4 (424.9) | | 25.9 (29.8) | |
| 2010 | 8.29 (4.6) | 5.9 (2.7) | | 8.6 (4.7) | |
| 2014 | 6.9 (6.4) | 14.1 (9.2) | | 6.9 (4.9) | |
| ***Uganda*** | | | | | |
| 2000 | 1.2 (0.4) | 2.8 (1.1) | | 1.9 (0.7) | |
| 2006 | 2.6 (0.9) | 26.0 (33.8) | | 2.2 (0.7) | |
| 2011 | 2.2 (0.7) | 10.6 (6.7) | | 3.5 (1.5) | |

**
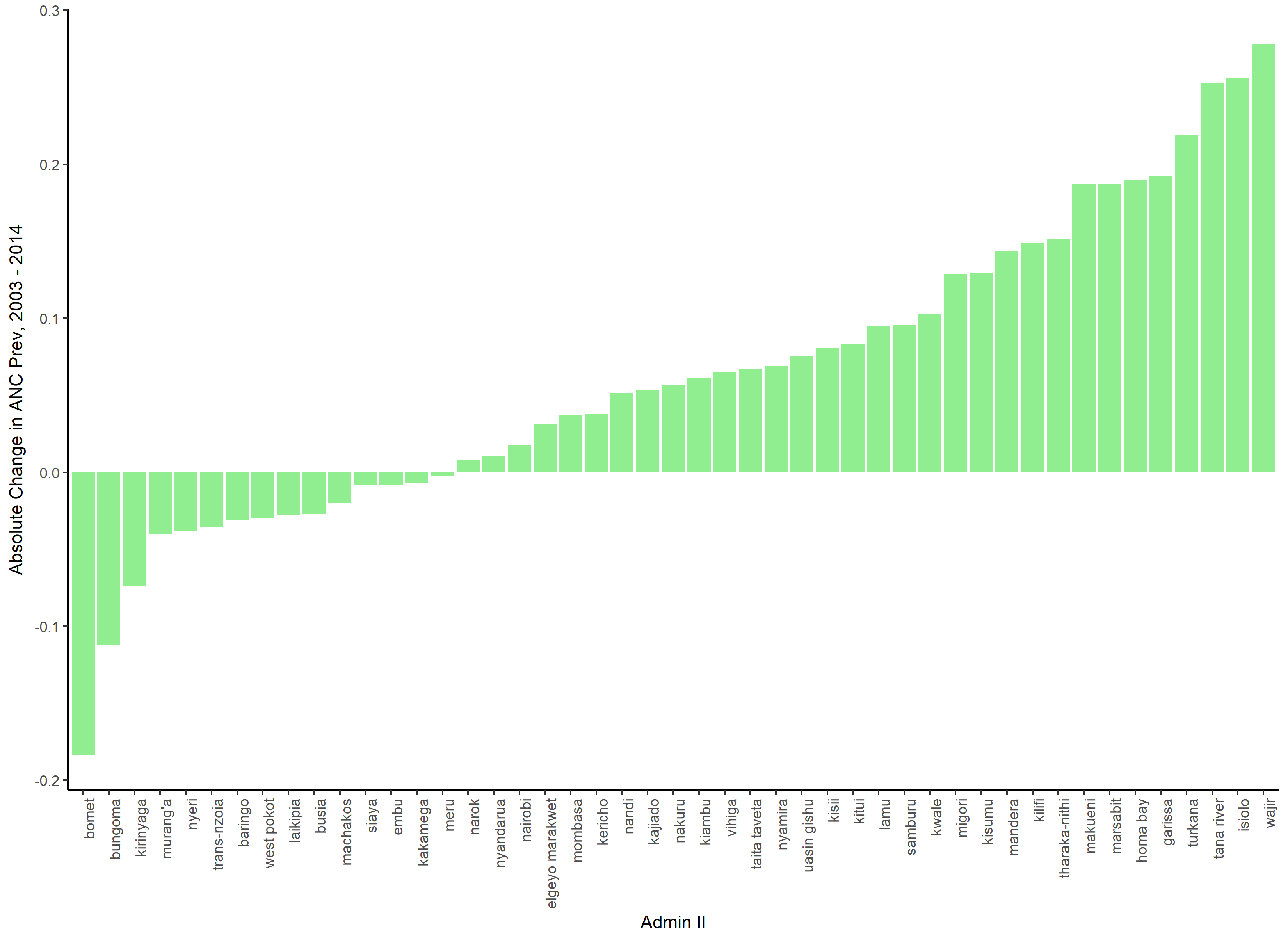
Figure A-1a.** Absolute change in **a)** 4+ antenatal care visits (green), **b)** postnatal care check-up within 48 hours (red), and **c)** skilled birth attendance (blue), Kenya DHS data, 2003 – 2014, ordered by administrative II unit


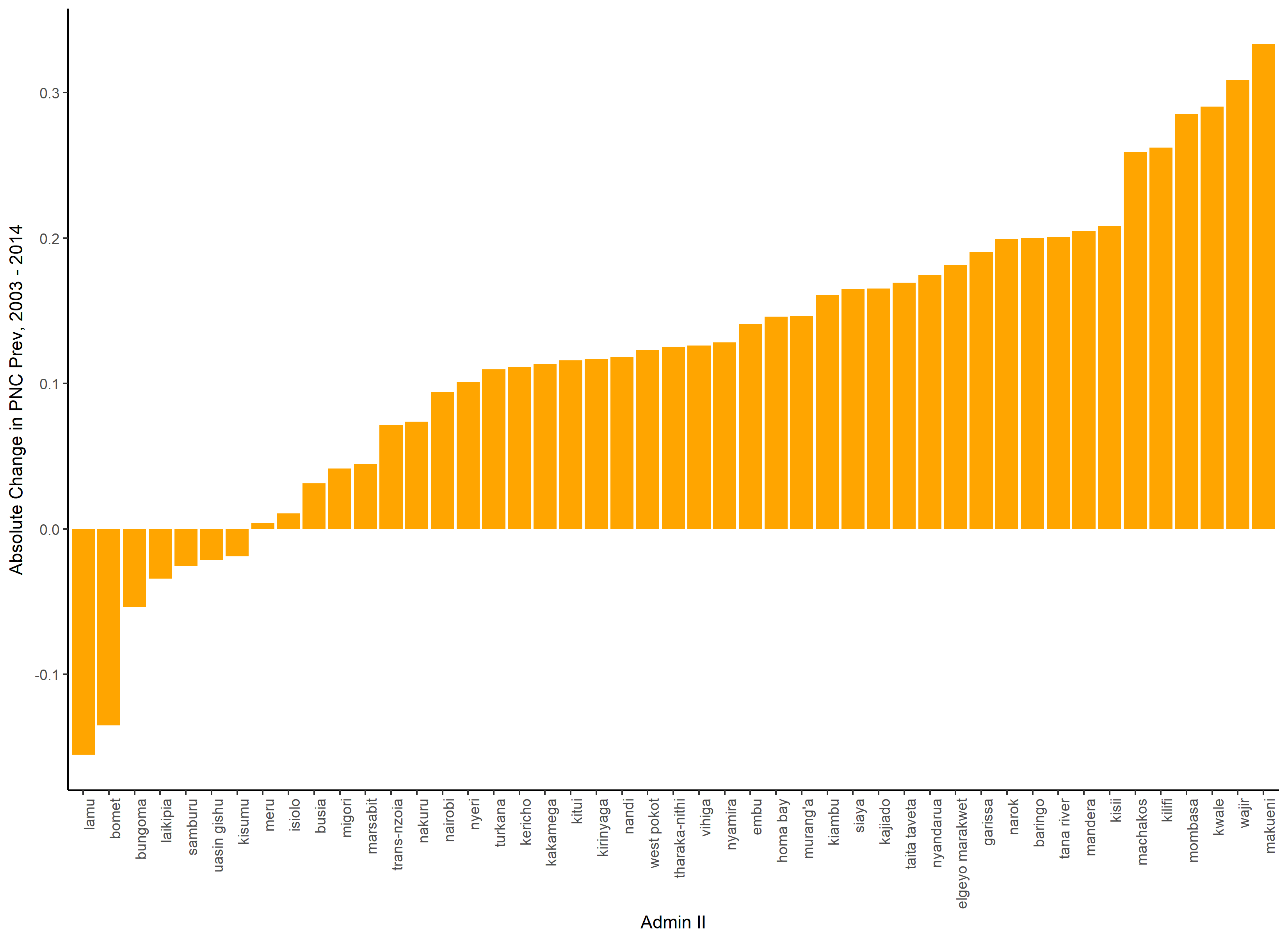


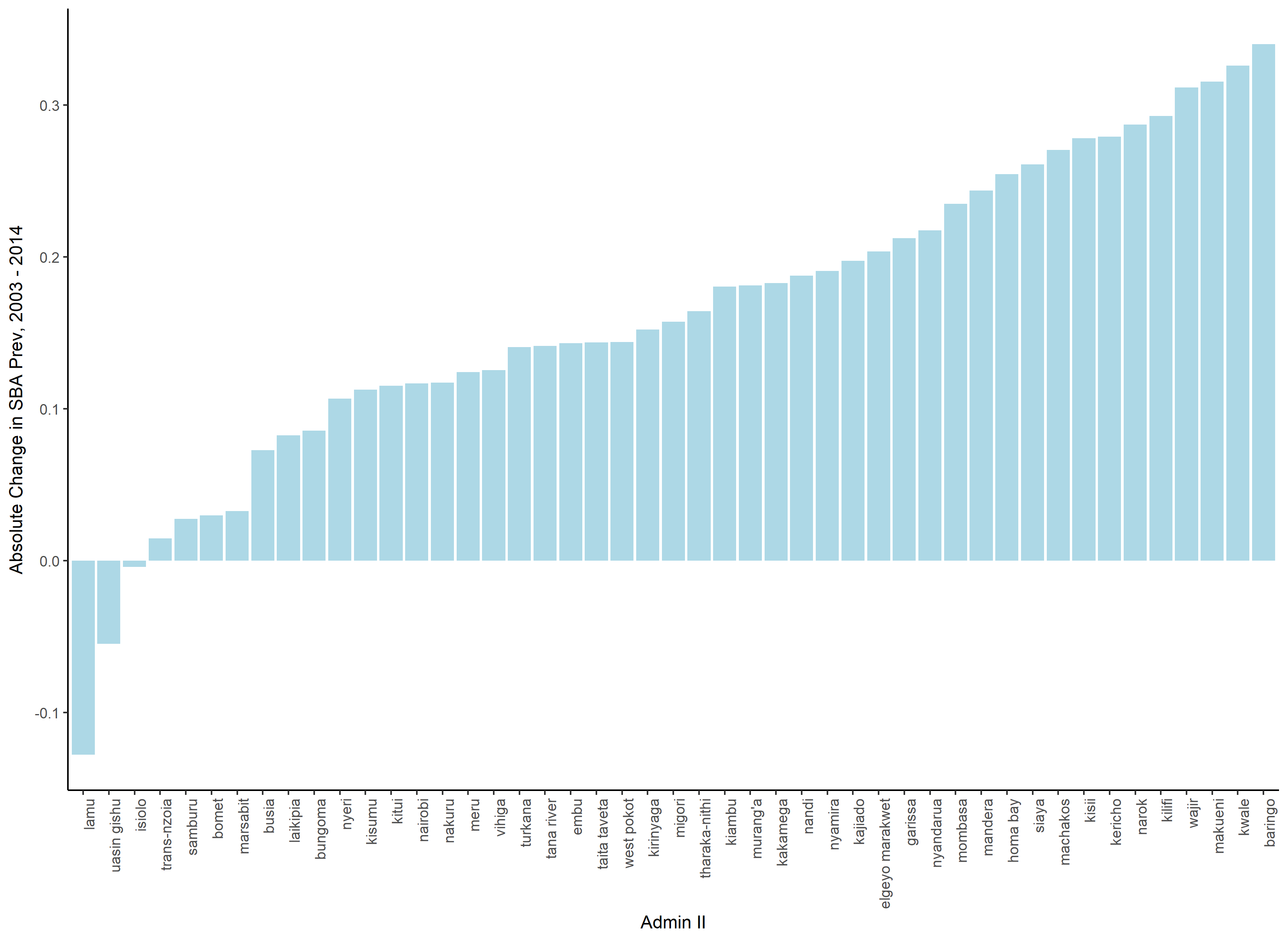


**
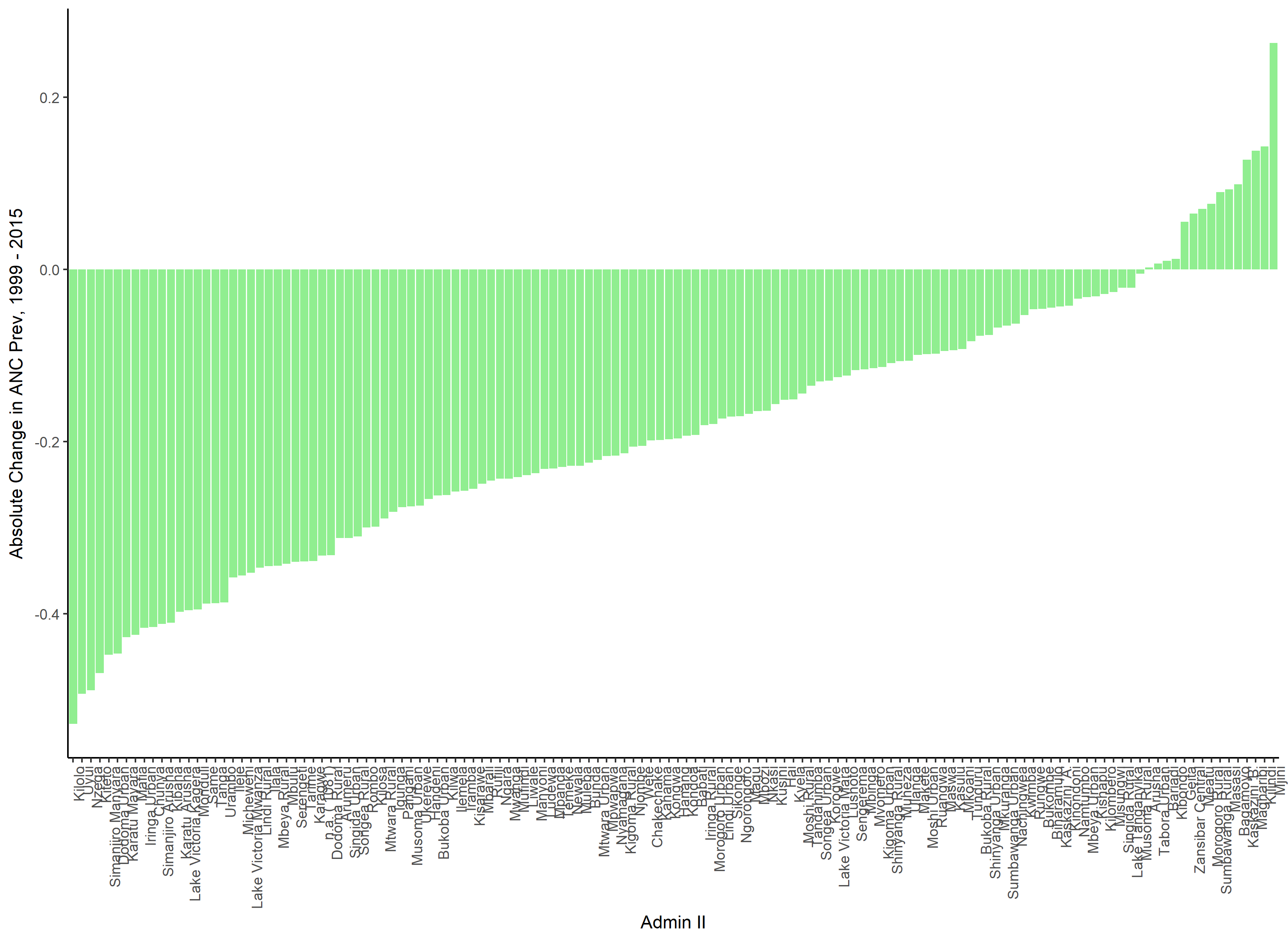
Figure A-1b.** Absolute change **a)** 4+ antenatal care visits (green), **b)** postnatal care check-up within 48 hours (red), and **c)** skilled birth attendance (blue), Tanzania DHS data, 1999 – 2015, ordered by administrative II unit


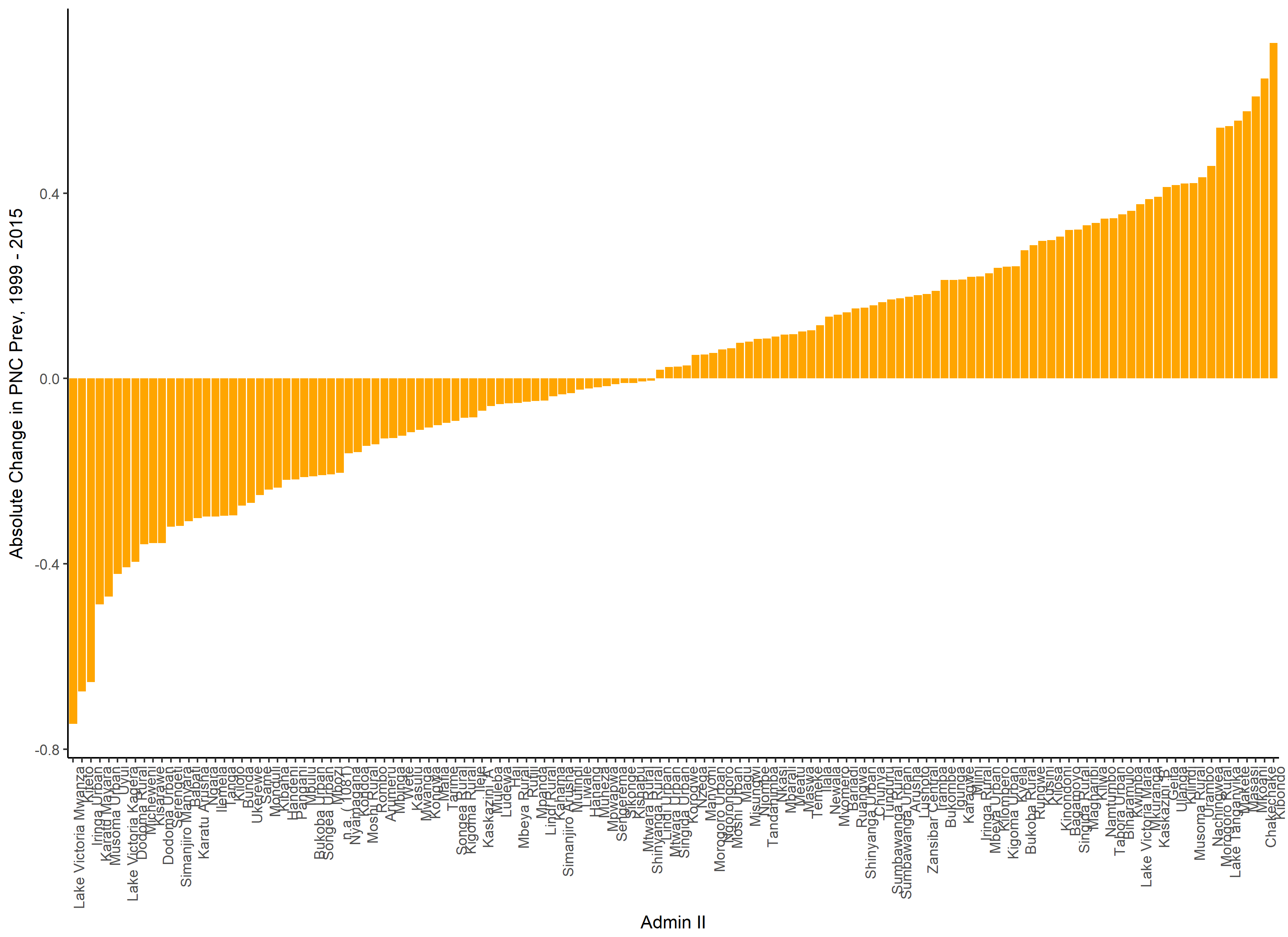


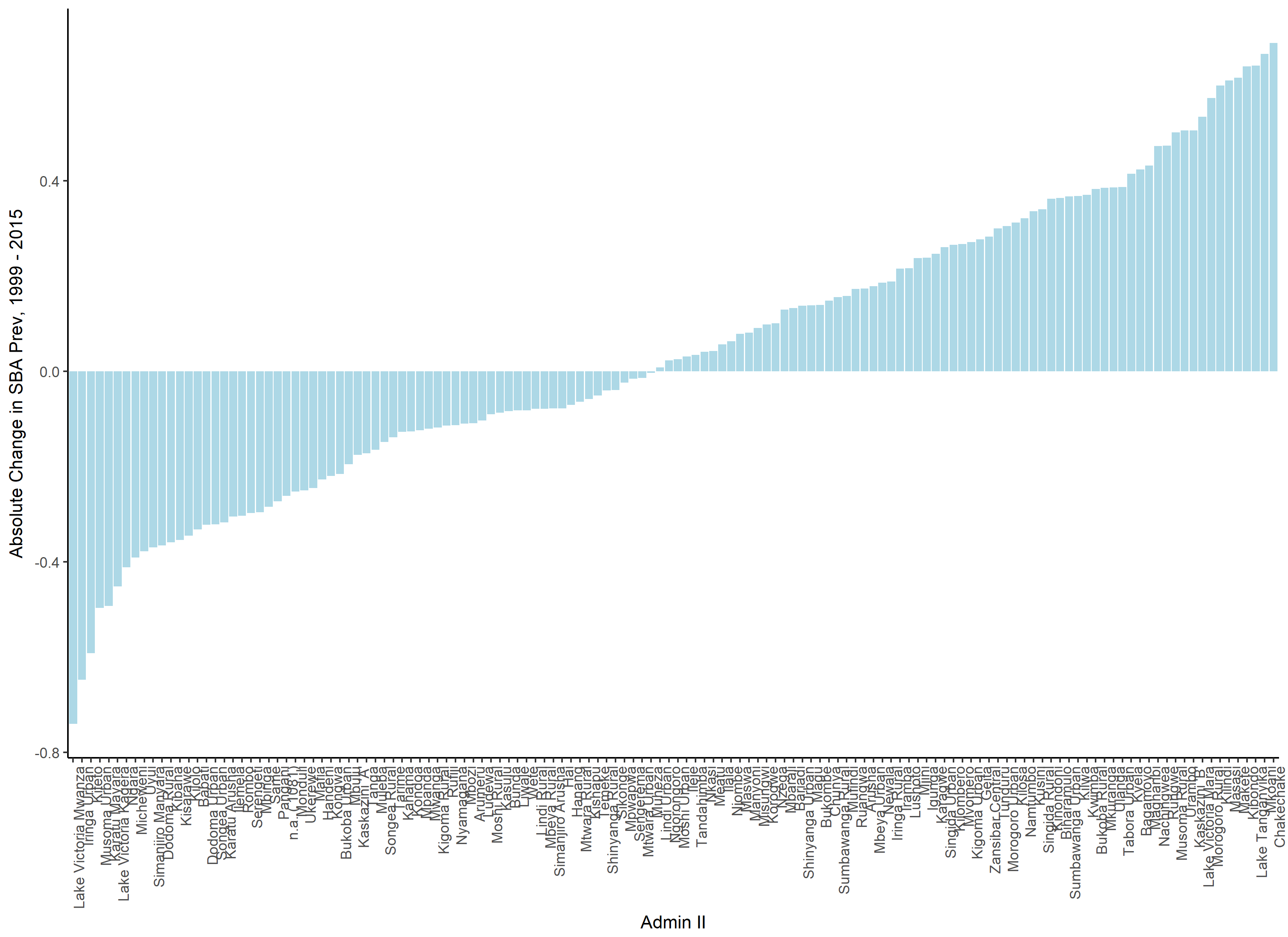
**Figure A-1c.** Absolute change **a)** 4+ antenatal care visits (green), **b)** postnatal care check-up within 48 hours (red), and **c)** skilled birth attendance (blue), Rwanda **
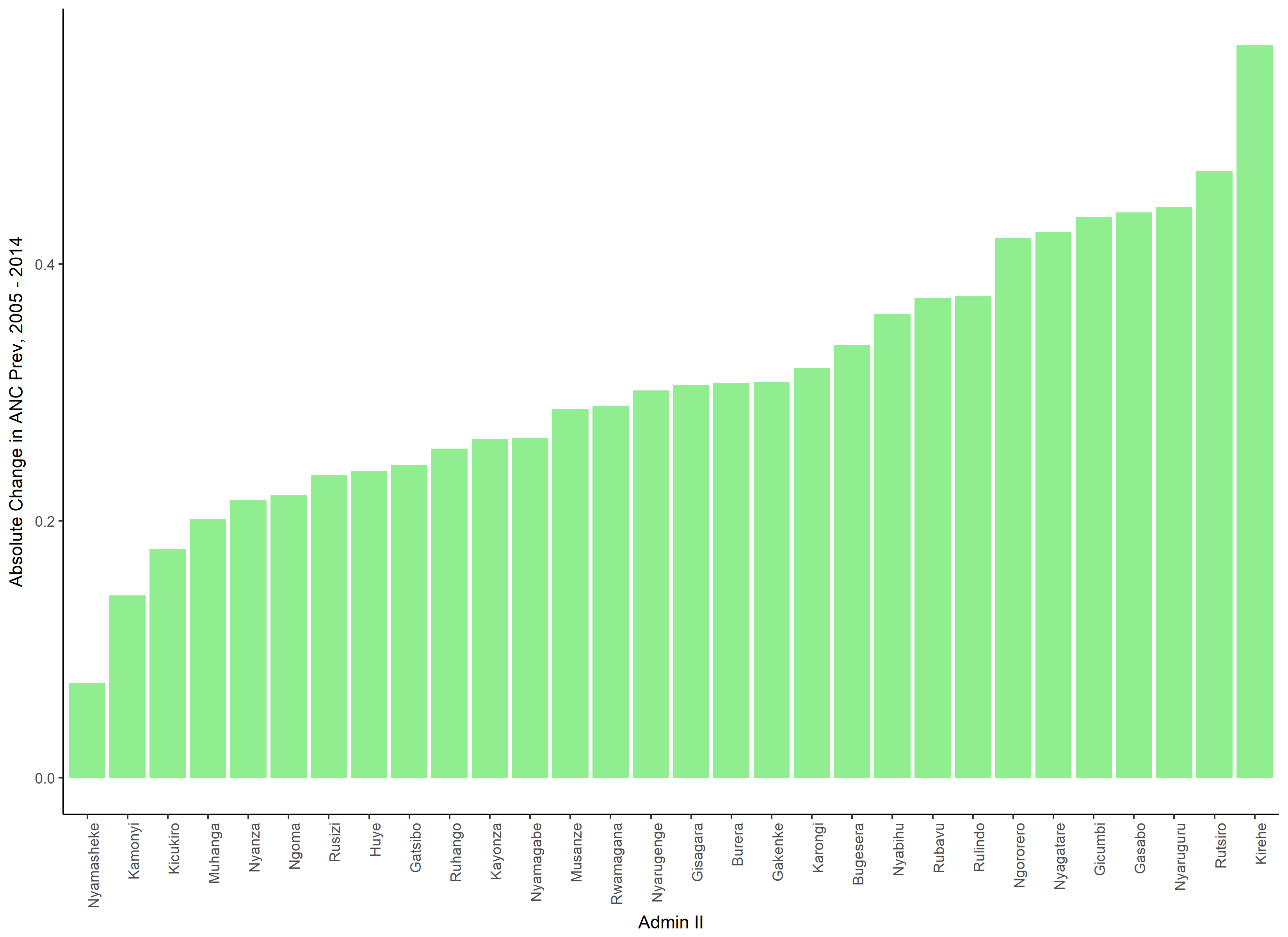
**DHS data, 2005 – 2014, ordered by administrative II unit


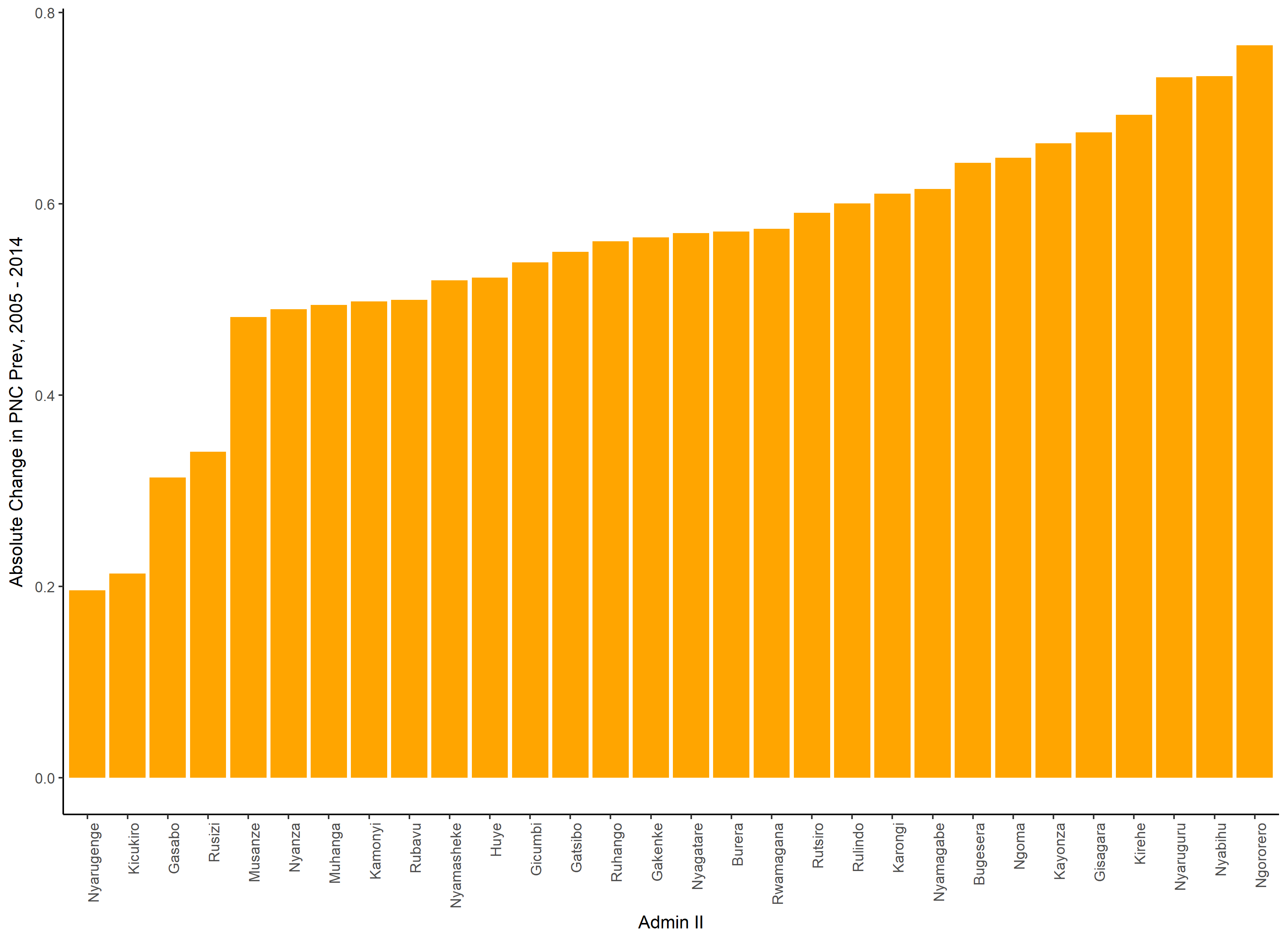


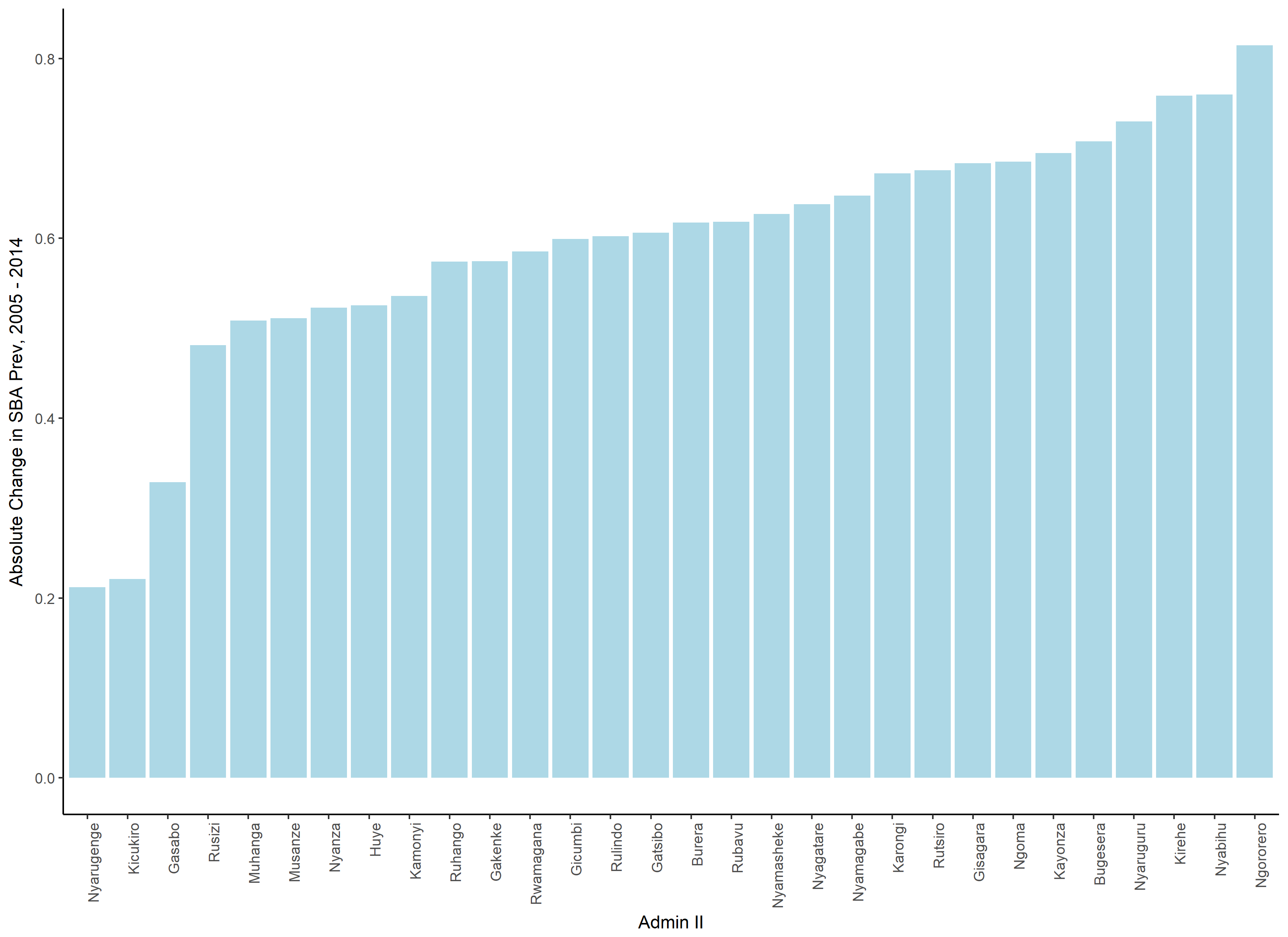
**Figure A-1d.** Absolute change in **a)** 4+ antenatal care visits (green), **b)** postnatal care check-up within 48 hours (red), and **c)** skilled birth attendance (blue), Uganda **
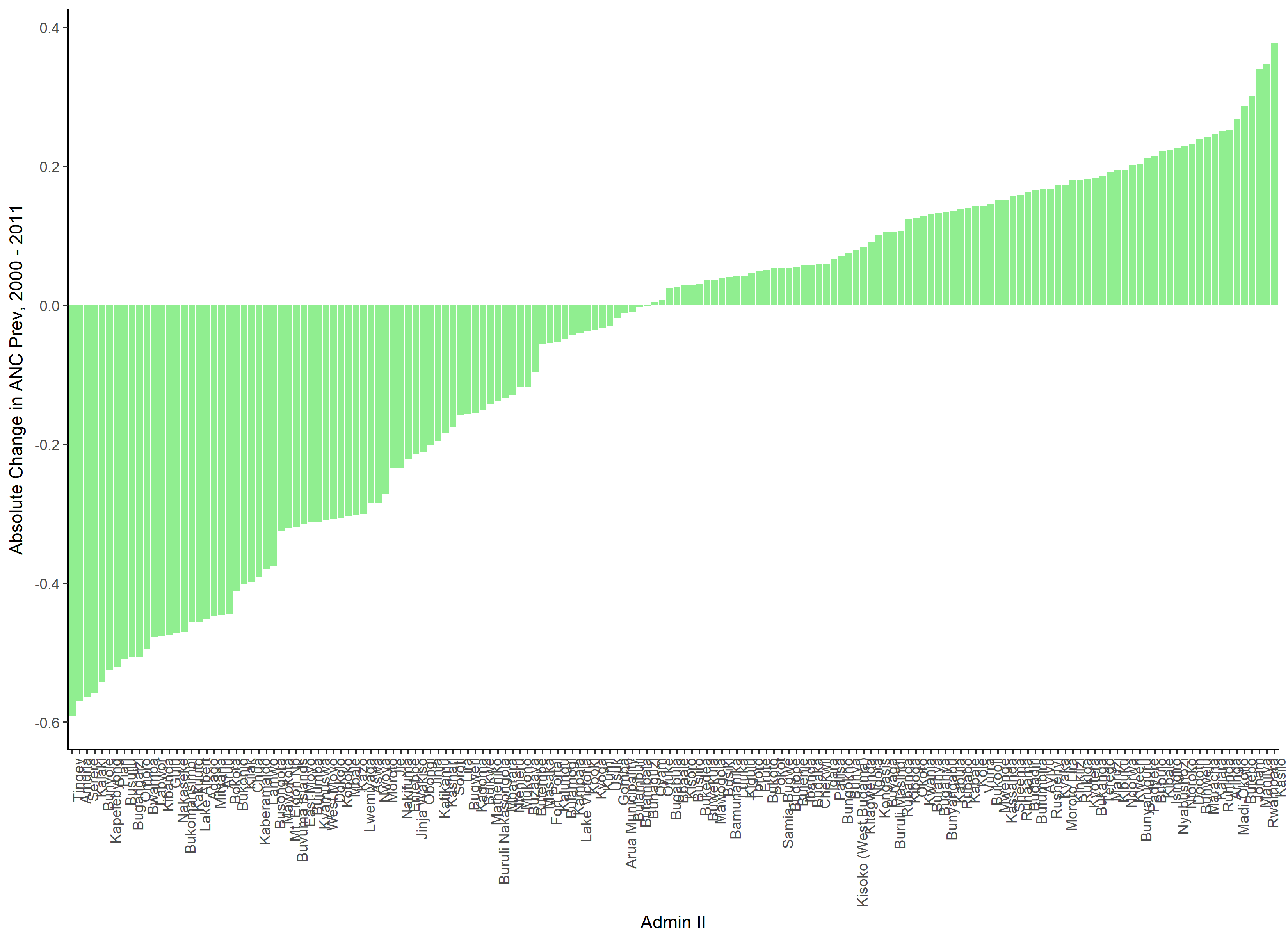
**DHS data, 2000 – 2011, ordered by administrative II unit


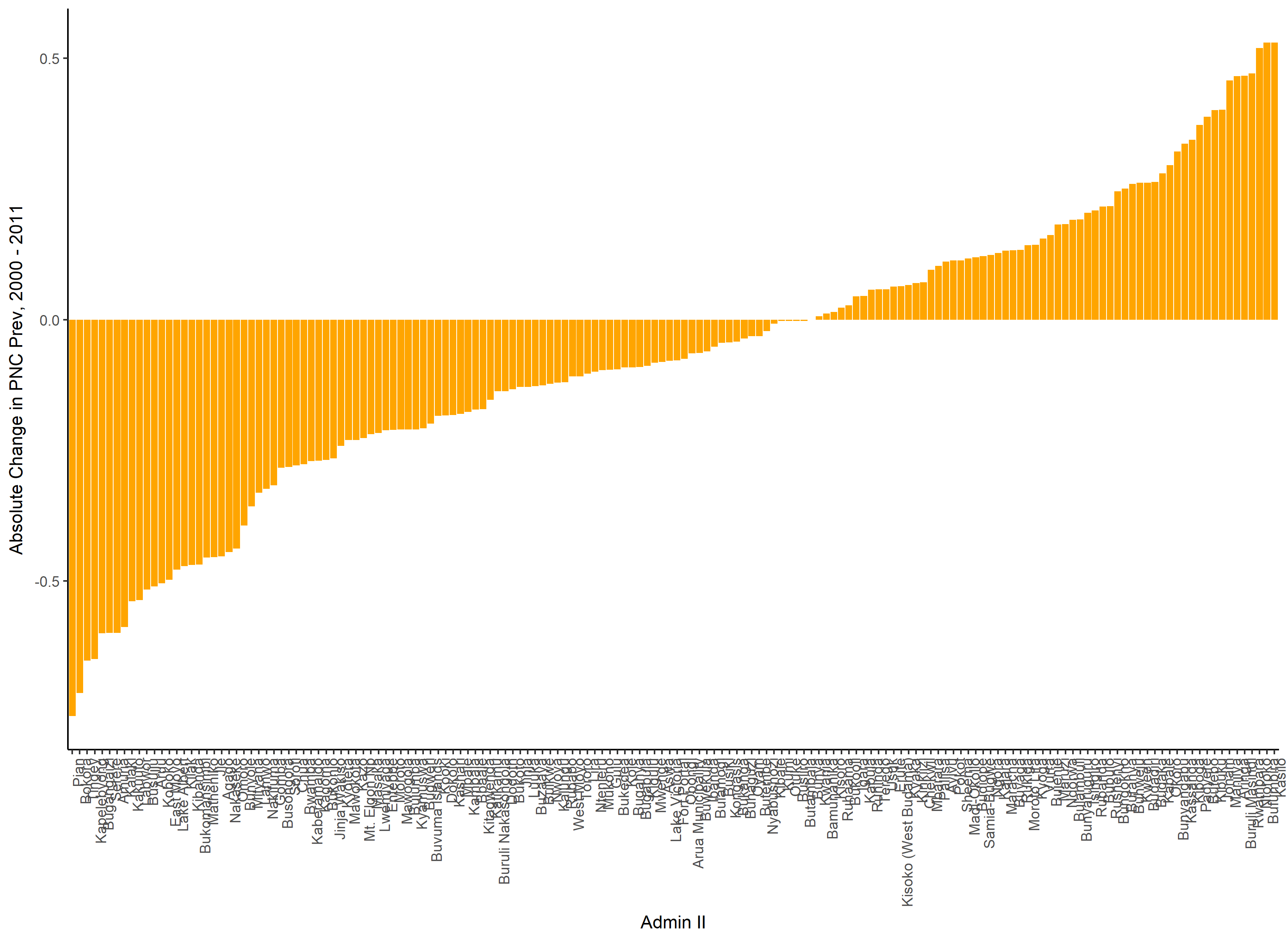


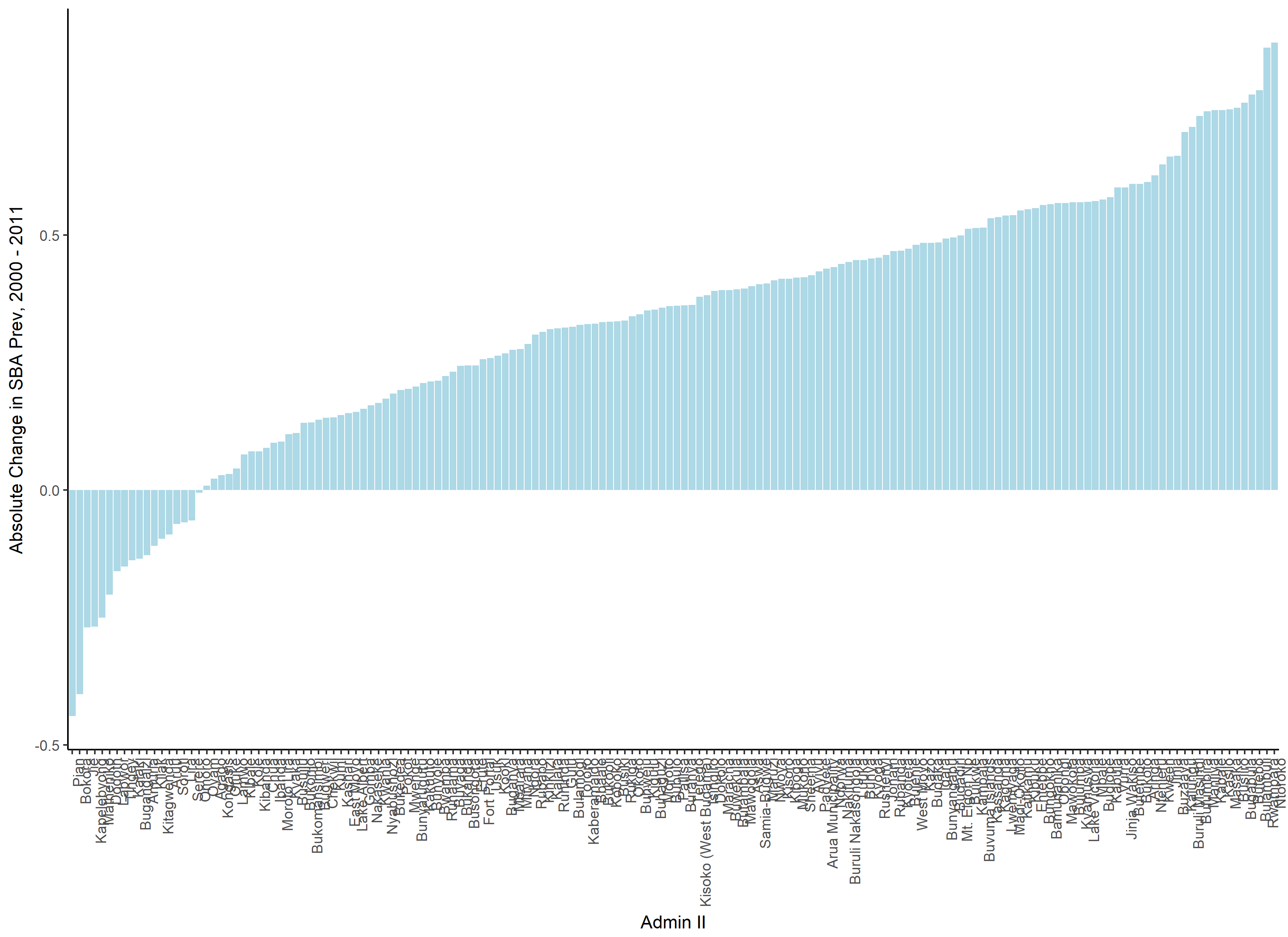

Supplement: Supplementary file 1 — Appendix. Supplementary information containing 1) unadjusted logistic regression results; 2) model fit, mean posterior estimates and hyperparameters; and 3) ordered absolute change in indicators by administrative II unit. (DOCX 1505 kb) [file 12889_2018_6241_MOESM1_ESM.docx]
